# Supplementary material for: 60 cm2 perovskite-silicon tandem solar cells with an efficiency of 28.9% by homogeneous passivation
Source: Nat Commun. 2025 Sep 30;16:8672. doi: 10.1038/s41467-025-63673-y (PMC12485175; doi:10.1038/s41467-025-63673-y)
Supplement: Supplementary file 1 — Supplementary Information [file 41467_2025_63673_MOESM1_ESM.pdf]

Supplementary Information for

60 cm² perovskite-silicon tandem solar cells with an efficiency of 28.9% by homogeneous passivation

Artuk *et al.*

Corresponding authors: Kerem Artuk, kerem.artuk@epfl.ch; Christian M. Wolff, christian.wolff@epfl.ch

The PDF file includes:

Figs. S1 to S39
Table S1 to S2

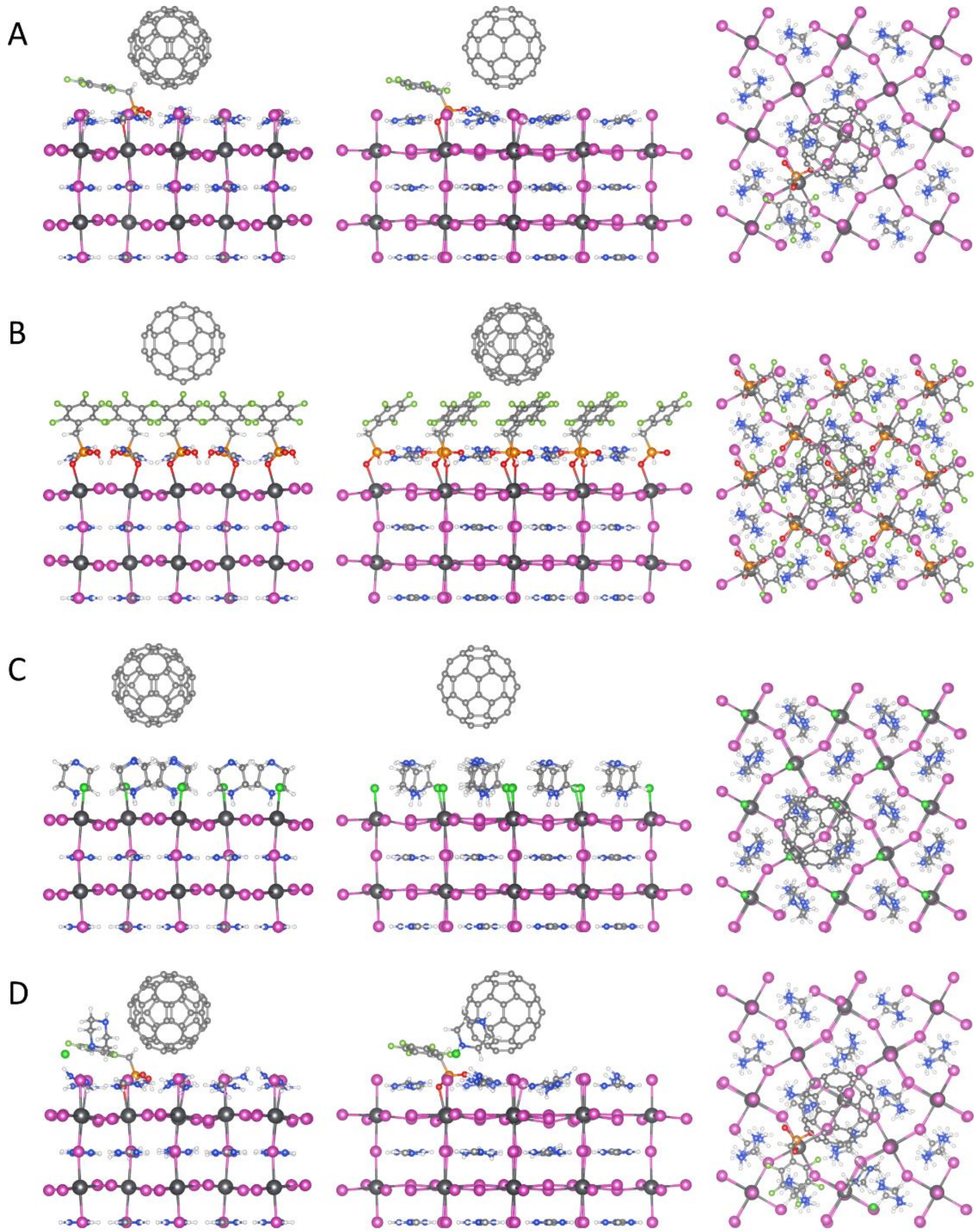

Figure S1. Side and top views of perovskite···C_{60} interfaces with (A) 12.5% and (B) 100% surface substitution of IPb-I by IPb-$OPO_2HCH_2C_6F_5$, (C) 100% surface substitution of FAI by $HNC_4H_8NH_2$·Cl, (D) 12.5% surface substitution of IPb-I by IPb-$OPO_2HCH_2C_6F_5$ with one $HNC_4H_8NH_2$·Cl ion pair in the vicinity of a I···C_{60} contact.

	E_b, eV	Distance, Å	Bader charge on C_{60}, electrons
pristine	-0.86	I···C_{60}: 3.7 (×2)	-0.05
IPb-$OPO_2HCH_2C_6F_5$, 12.5%	-0.86	I···C_{60}: 3.1, F···C_{60}: 3.3, O···C_{60}: 3.6 (×2)	-0.03
IPb-$OPO_2HCH_2C_6F_5$, 100%	-0.96	F···C_{60}: 3.1 (×3), 3.3 (×2)	+0.06
$HNC_4H_8NH_2$·Cl, 100%	-0.38	N···C_{60}: 4.1 (×2), 4.2 (×2)	0.00
IPb-$OPO_2HCH_2C_6F_5$, 12.5%, $HNC_4H_8NH_2$·Cl, 12.5%	-0.99	I···C_{60}: 3.2, F···C_{60}: 3.3, O···C_{60}: 3.5 (×2), N···C_{60}: 3.3	-0.04

Table S1. Binding energy (E_b, eV), distance of the N/O/F/I···C_{60} contact (Å), and Bader charge accumulation on C_{60} for the different perovskite surfaces.

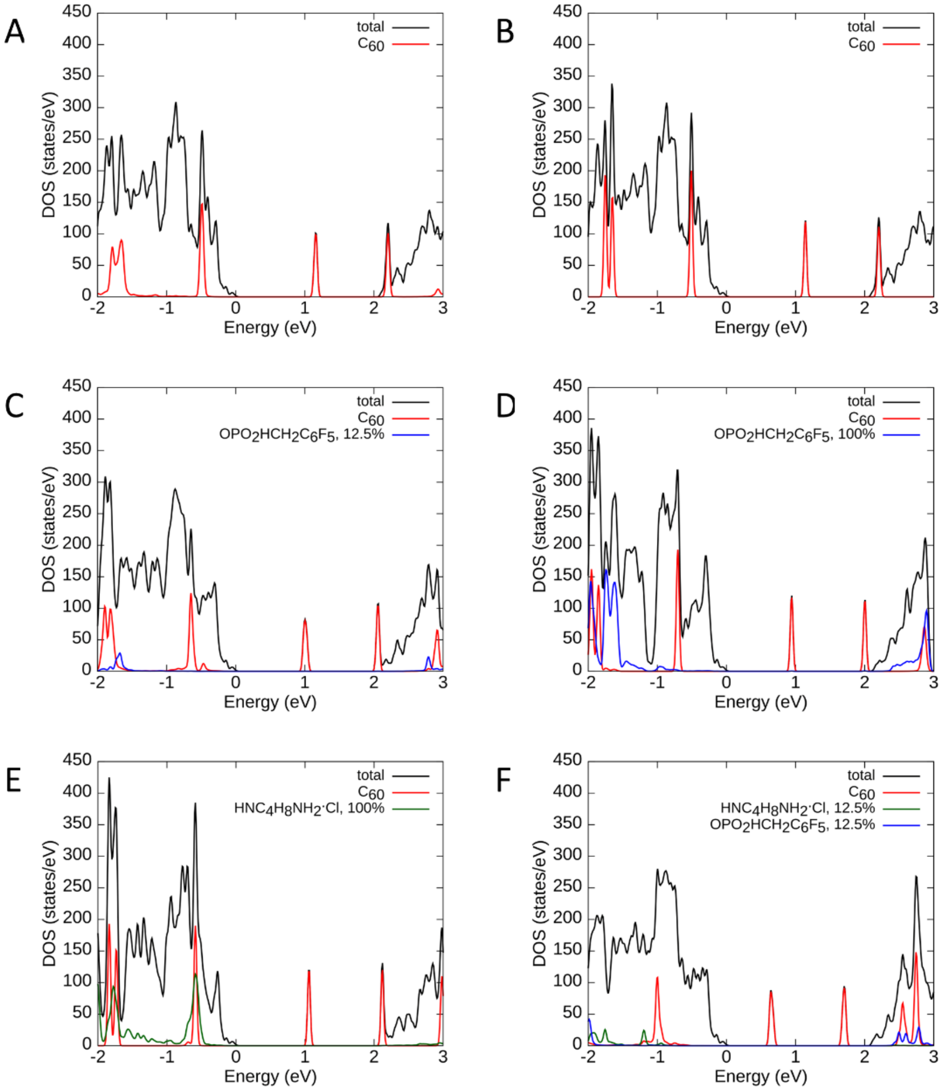

Figure S2. Density of states of perovskite···C_{60} interfaces: (A) pristine surface with 8.6 Å separation from C_{60} (negligible interaction), (B) pristine surface with 3.7 Å separation from C_{60} (maximum interaction), (C) 12.5% and (D) 100% surface substitution of IPb-I by IPb-$OPO_2HCH_2C_6F_5$, (E) 100% surface substitution of FAI by $HNC_4H_8NH_2$·Cl, (F) 12.5% surface substitution of IPb-I by IPb-$OPO_2HCH_2C_6F_5$ with one $HNC_4H_8NH_2$·Cl ion pair. The resolution of the k-grid was doubled for the density of states calculations and a Gaussian smearing of 0.002 Ry was applied. The overestimation of the band gap is mainly due to negligence of the spin-orbit coupling (computationally too expensive due to the large sizes of the considered systems).

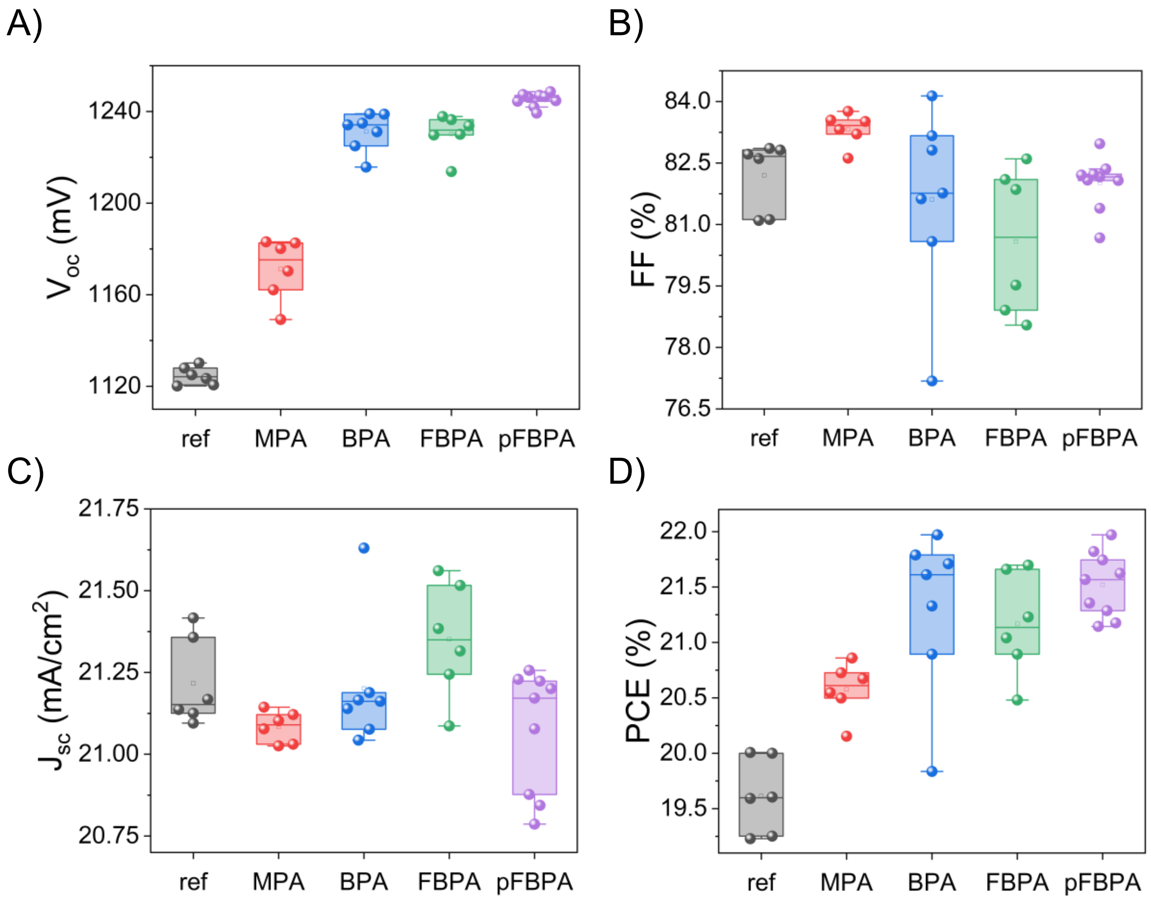

Figure S3. Device performance - a) V_{OC}, b) FF, c) J_{SC}, d) PCE of single junction devices with different phosphonic-acid derivatives at the perovskite/C_{60} interface.

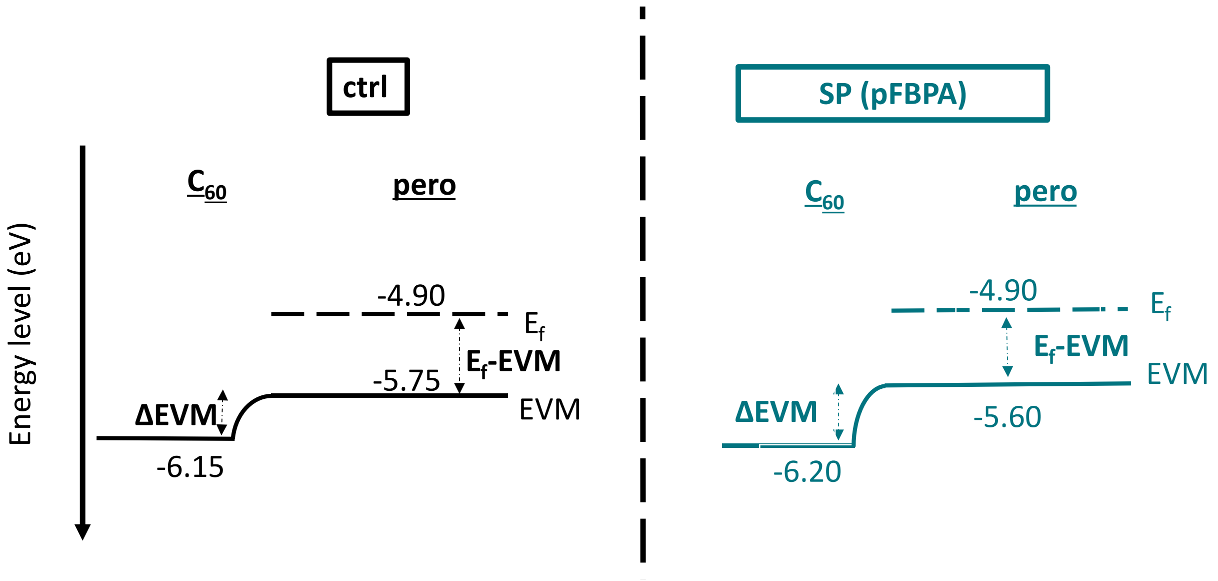

Figure S4. Band diagram control and surface passivation (pFBPA) extracted from UPS measurements.

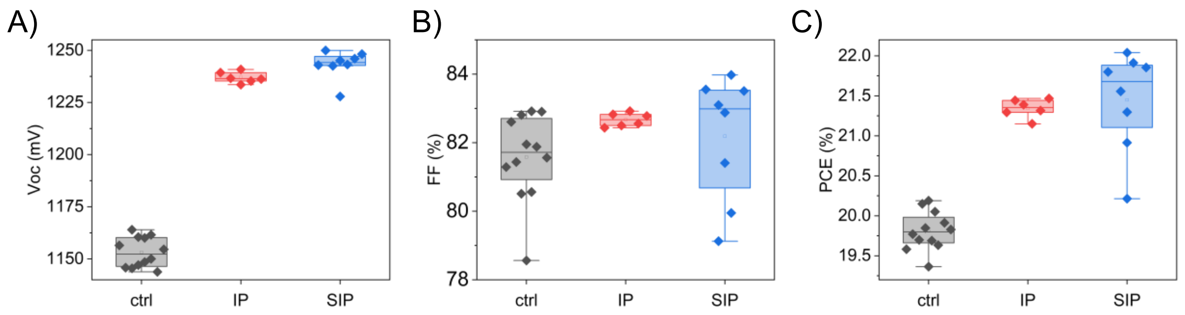

Figure S5. Device performance - a) V_{OC}, b) FF, c) PCE of single junction devices (without antireflective coating on the glass-side) with different control, IP, and SIP surface treatments at the perovskite/C_{60} interface.

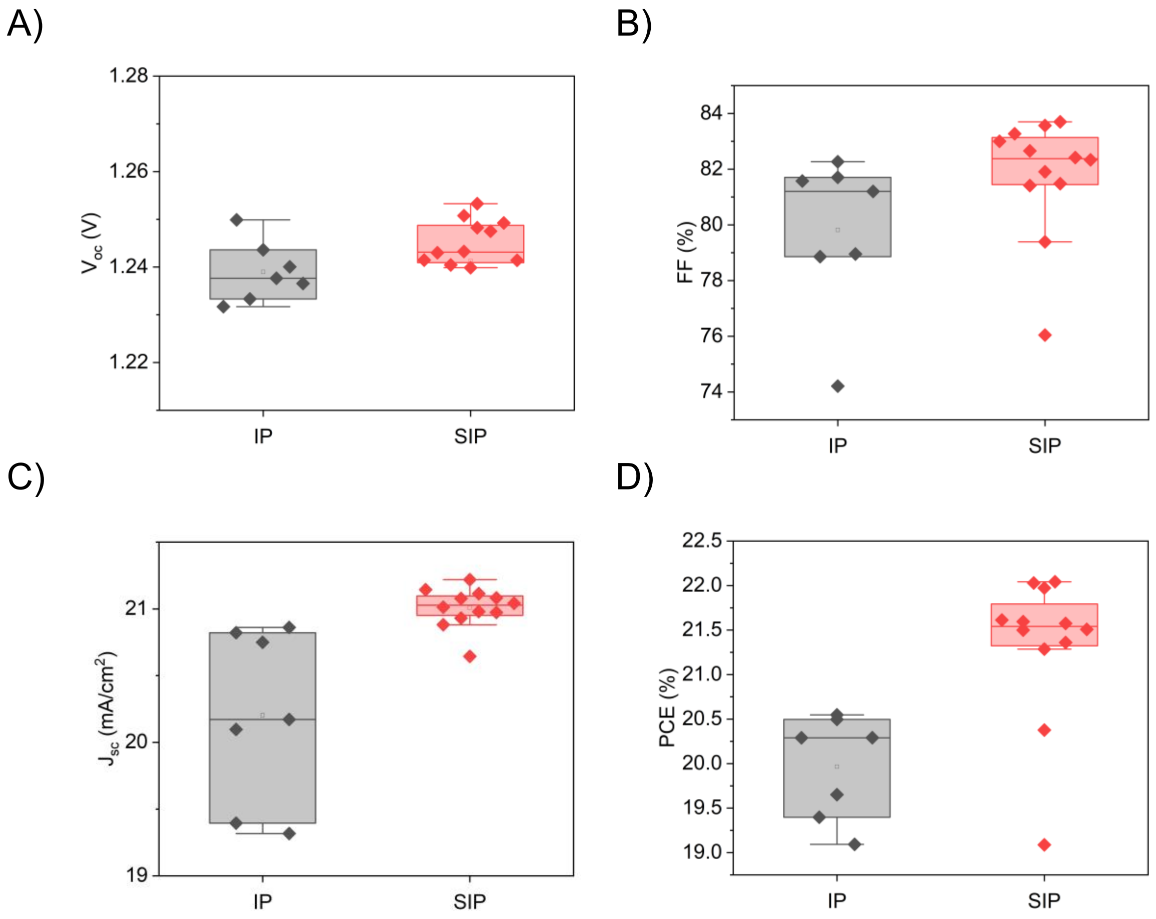

Figure S6. Device performance - a) V_{OC}, b) FF, c) J_{SC}, d) PCE in another batch of single junction devices with IP and SIP treatments at the perovskite/C_{60} interface.

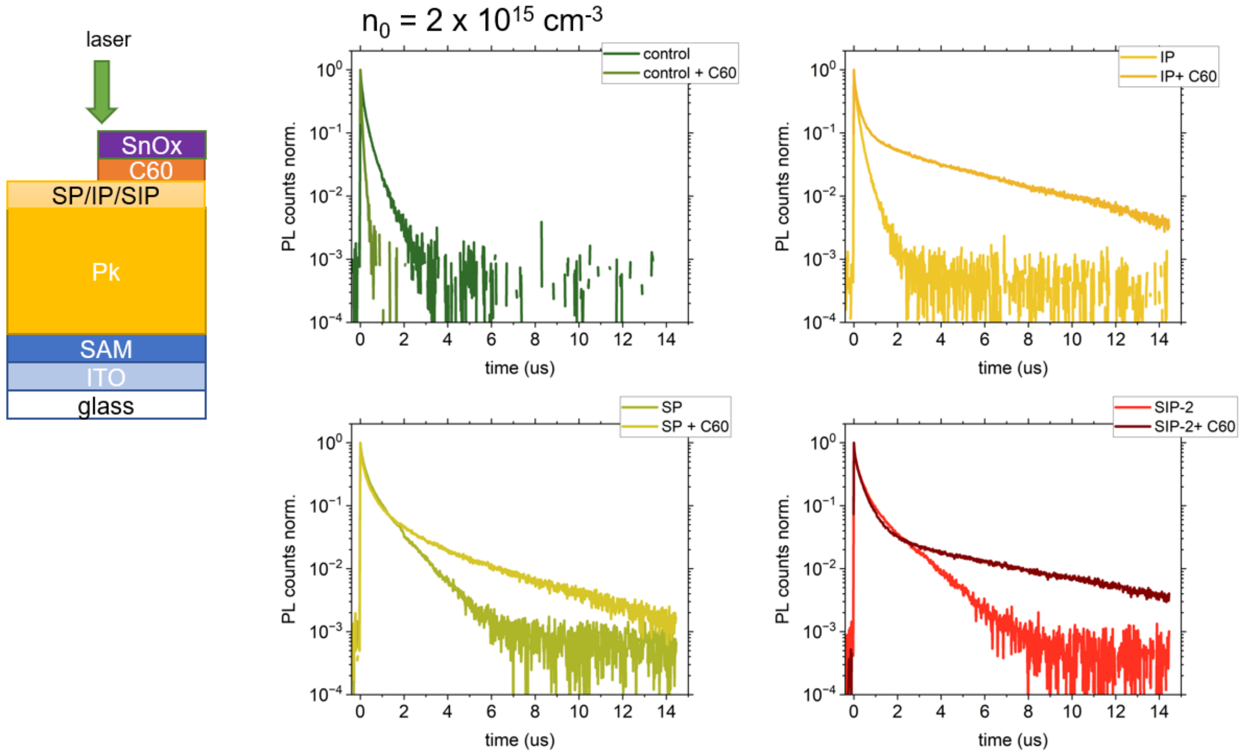

Figure S7. a) Schematics for the TRPL measurements (with and without ETL-stack) on half-cells with different passivation (control, IP, SP, and SIP).

Table.S2

Extracted t_3 lifetimes for the samples (on ITO/SAM) with different surface treatments (from the curves given in Fig.S7), with and without C_{60}/SnO_x.

	**Control**	**SP**	**IP**	**SIP**
T_3 without C_{60}/SnO_x	500 ns	1134 ns	423 ns	1396 ns
T_3 with C_{60}/SnO_x	93 ns	3271 ns	4693 ns	5296 ns

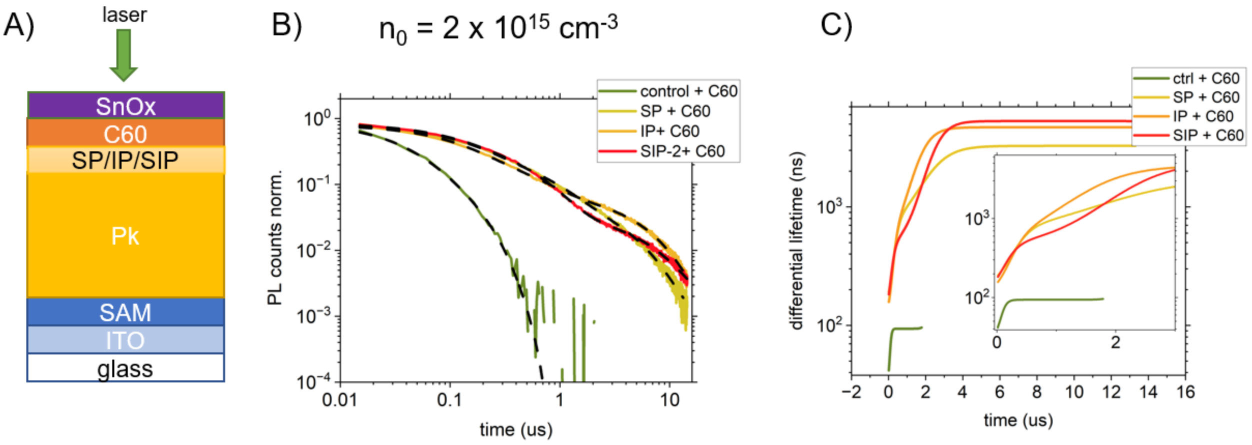

Figure S8. a) Schematics for the TRPL measurements. b) PL intensity for each condition and the fits (three exponential decay) show excellent matching with the experimental data. c) For the samples with ETL, differential lifetimes are extracted to compare charge extraction and t_3 lifetimes, inset: Closer look at the initial rise of the differential lifetime to compare charge extraction.

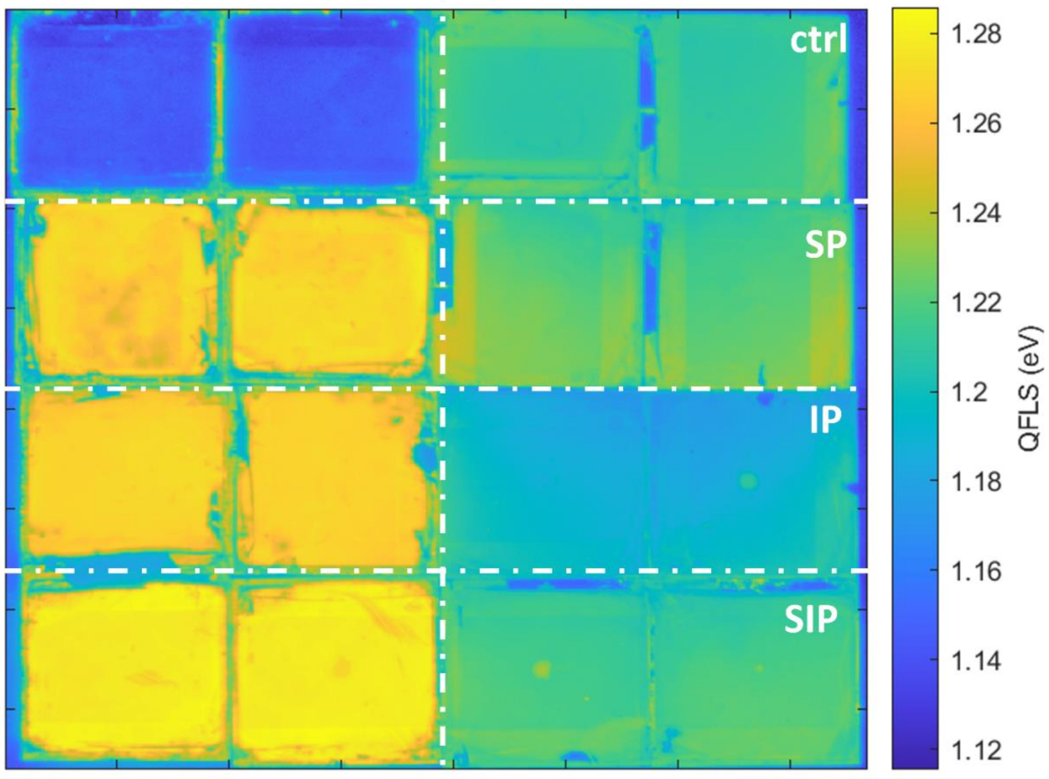

Figure S9. Wide-field PL imaging of QFLS of samples with control, SP, IP and SIP. The left side is with C_{60}/SnO_x stack and the right is without C_{60}/SnO_x. Each sample is 2.5x2.5 cm^2.

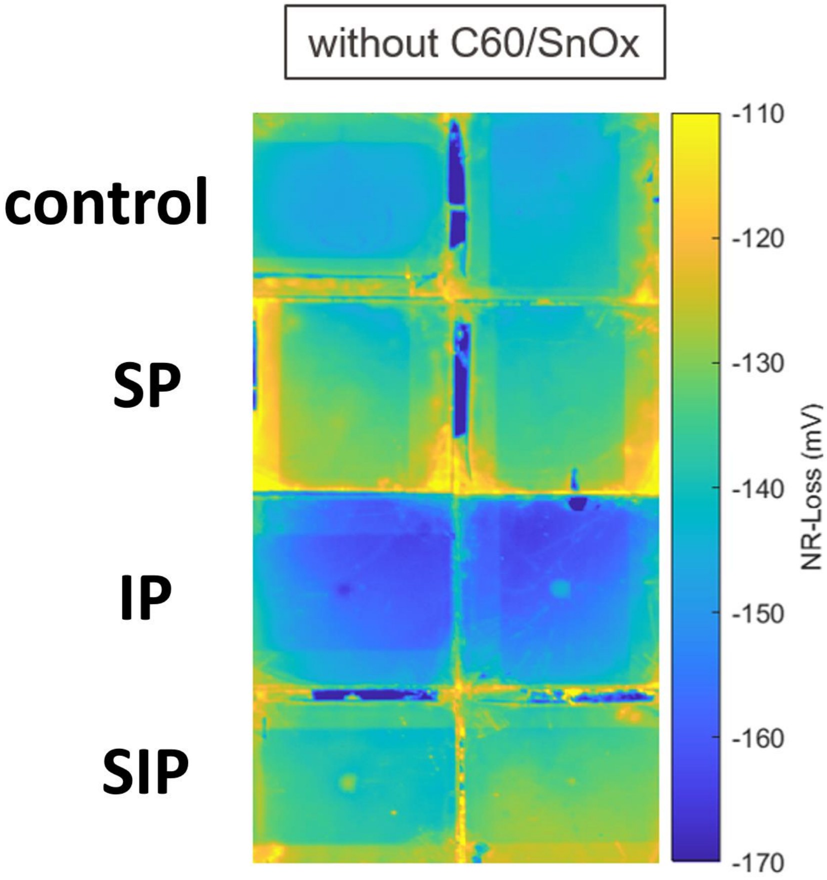

Figure S10. Wide-field PL imaging (non-radiative losses) of samples with control, SP, IP and SIP without C_{60}/SnO_x.

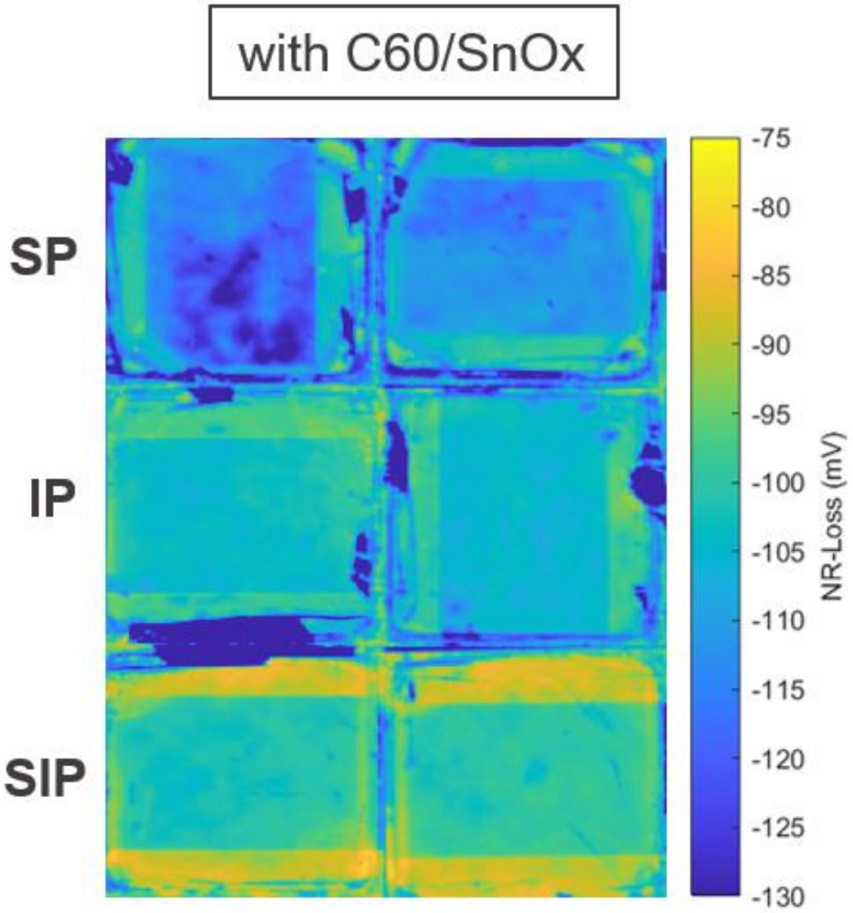

Figure S11. Wide-field PL imaging (non-radiative losses) of samples with control, SP, IP and SIP with C_{60}/SnO_x.

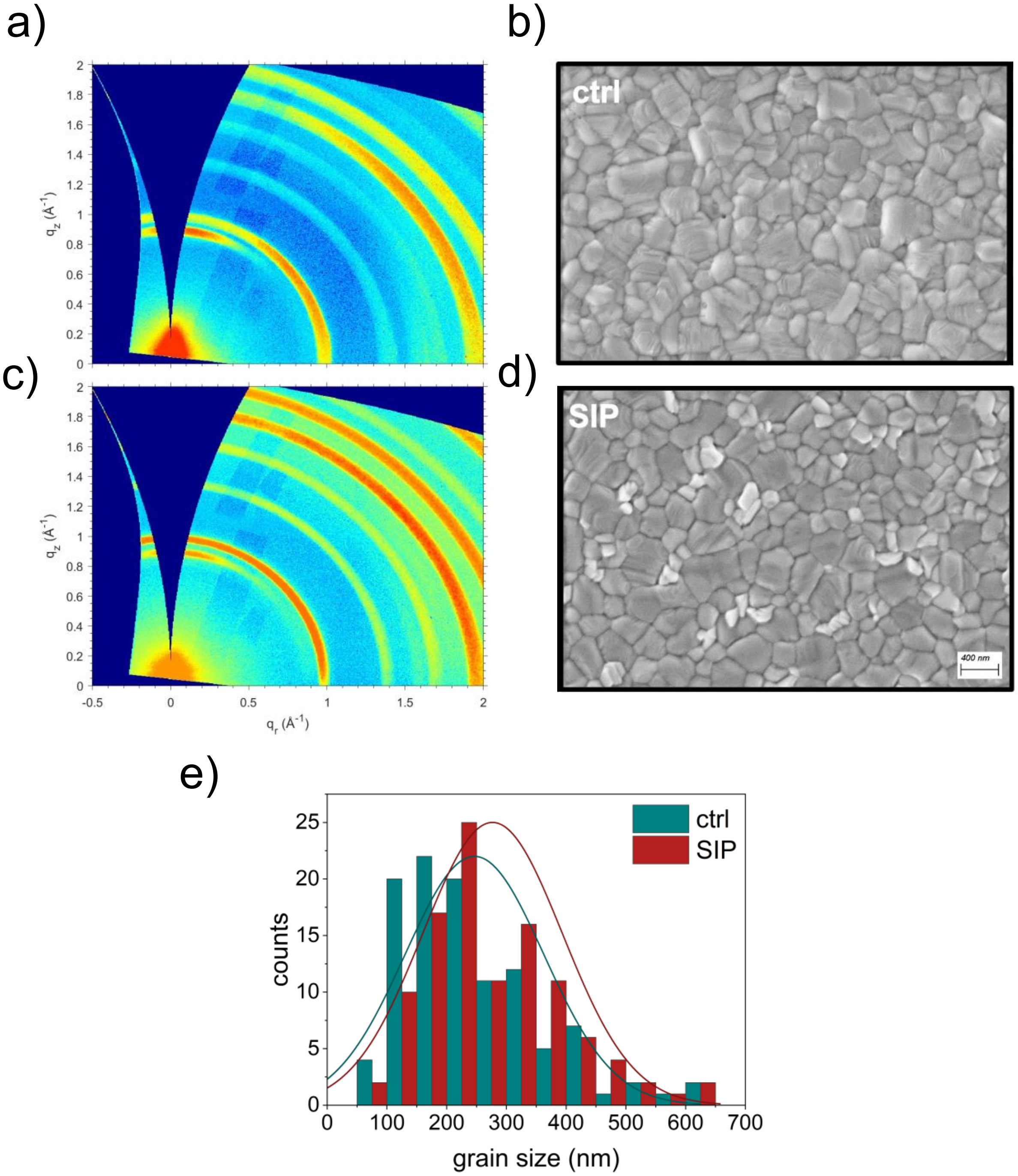

Figure S12. a) 2D GIWAXS maps for control samples, b) top-view SEM images for the control samples, c) 2D GIWAXS for SIP samples, d) top-view SEM image for SIP sample, e) grain size analysis of control and SIP samples

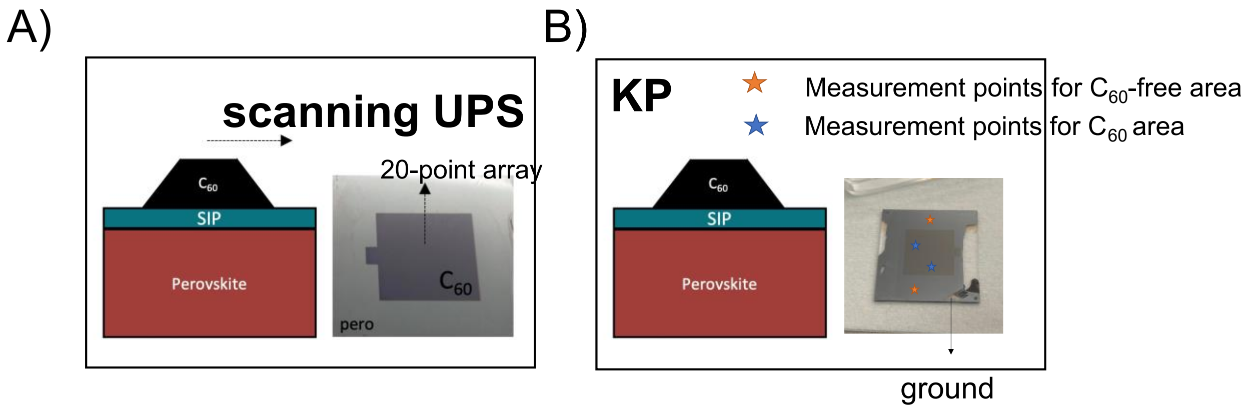

Figure S13. Representation of scanning UPS and KP measurements.

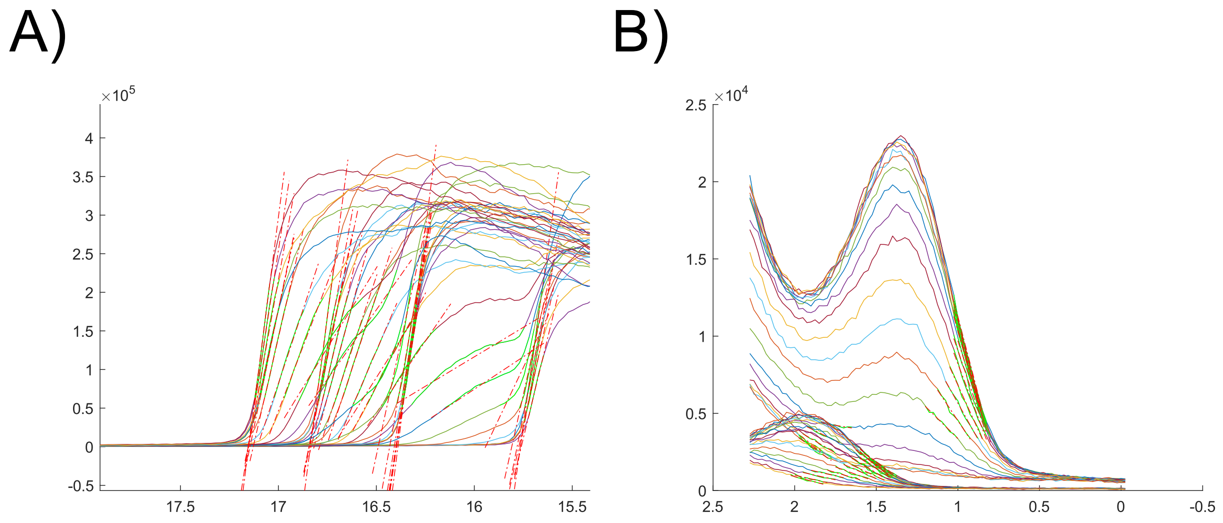

Figure S14. Example fits for the scanning UPS measurements – a) SECO, b) E_{VBM}.

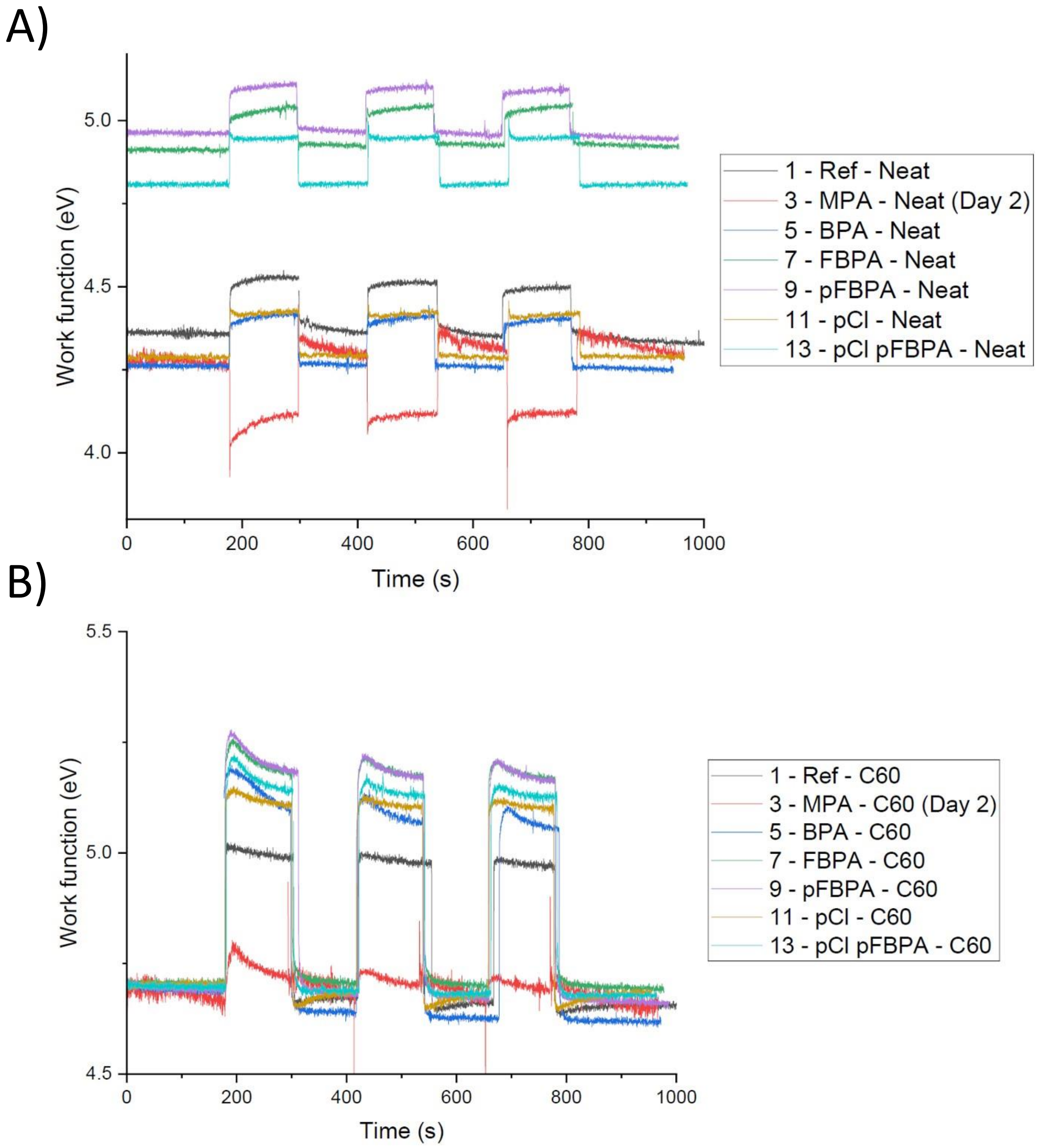

Figure S15. KP measurements for a) ITO/HTL/perovskite/passivation and b) ITO/HTL/perovskite/passivation/C60 under light and dark

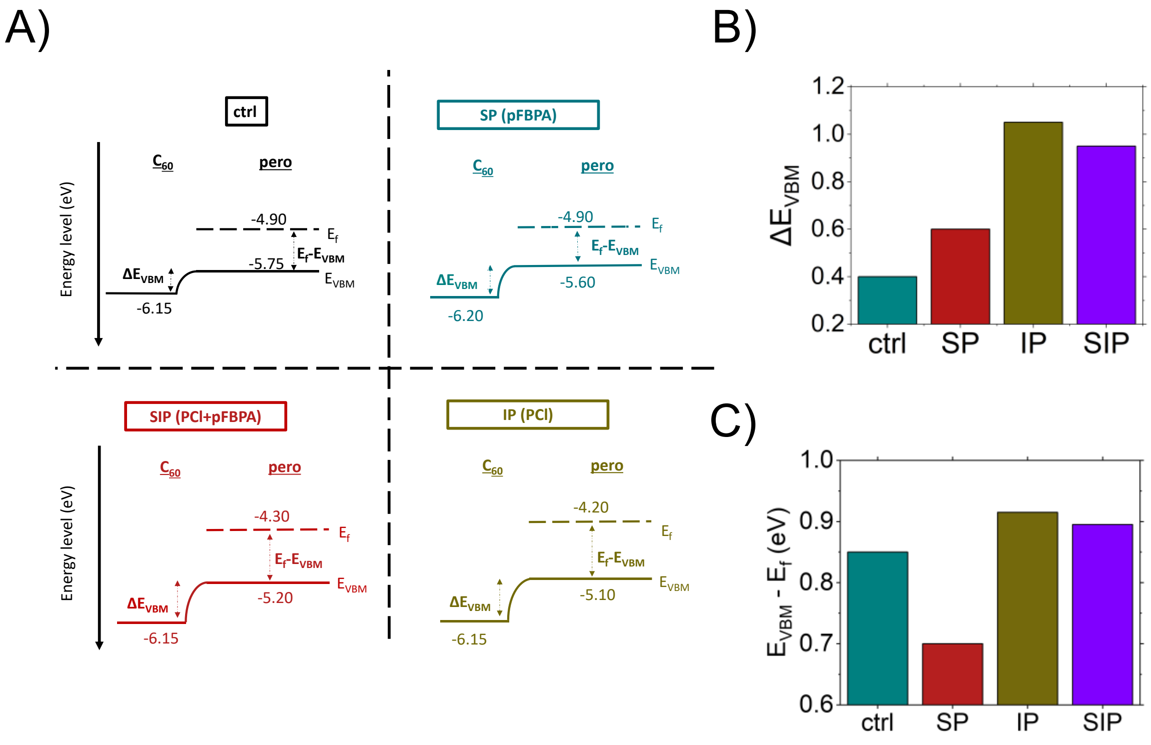

Figure S16. a) Band energetics extracted from scanning UPS measurements for control, IP, SP and SIP with extracted b) E_{VBM} offset between perovskite/C_{60} and c) the distance between from Fermi level to the E_{VBM} on perovskite with different surface treatments.

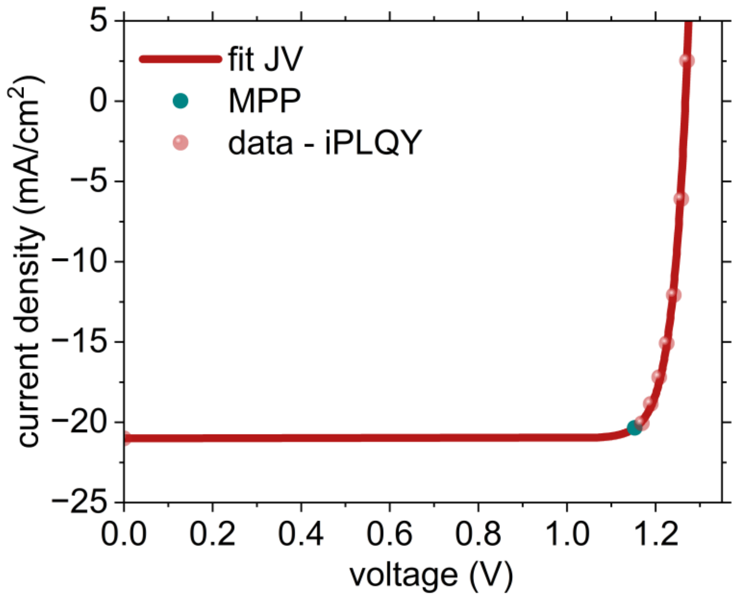

Figure S17. Intensity-dependent PLQY measurements for the single junction devices with SIP – pFF of 88.18%

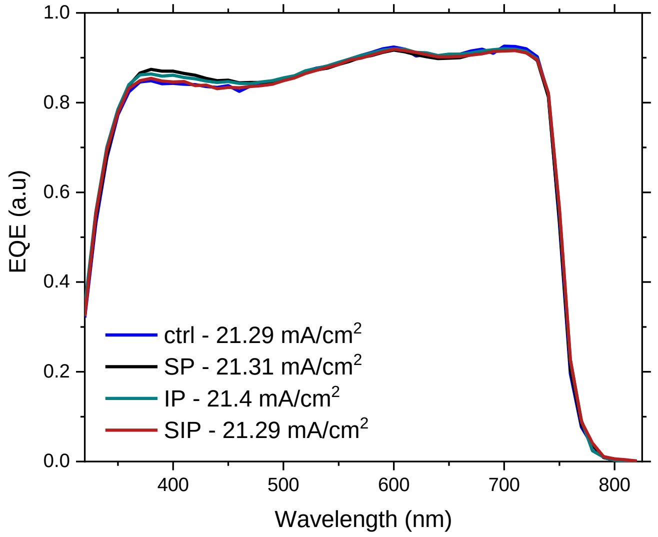

Figure S18. EQE results for control and devices with IP, SP, and SIP.

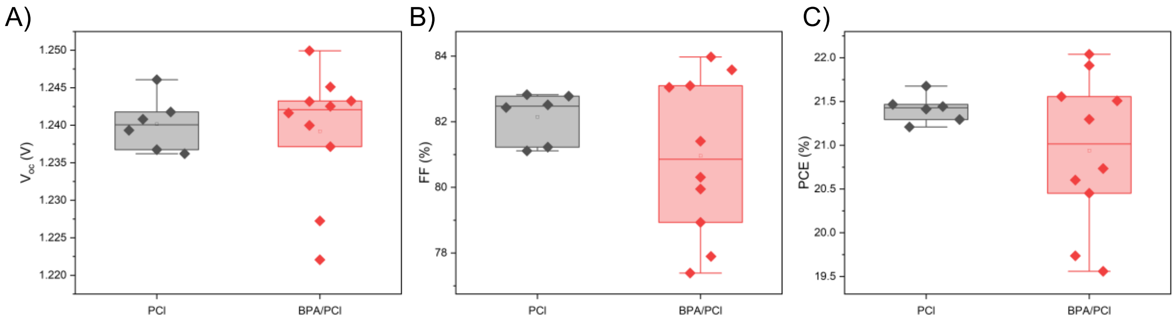

Figure S19. Device performance – a) V_{OC}, b) FF, c) PCE, of single junction devices with PCl or BPA/PCl the perovskite/C_{60}.

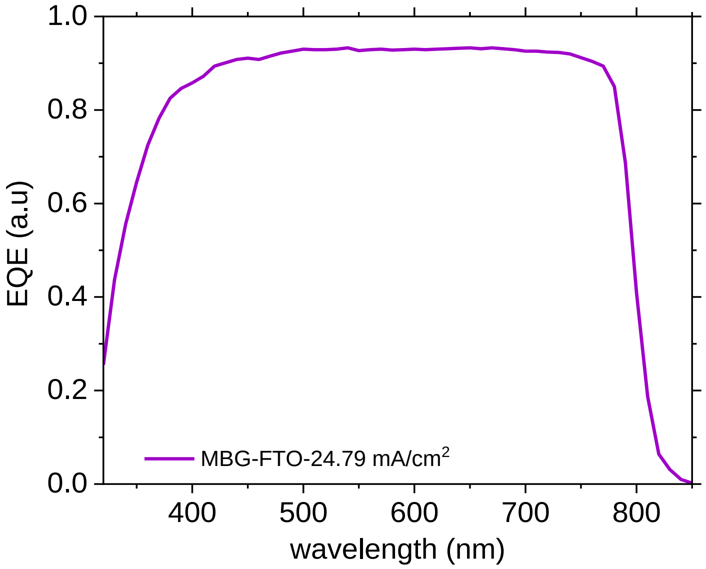

Figure S20. EQE of medium bandgap single junction devices with SIP on FTO substrates.

A)

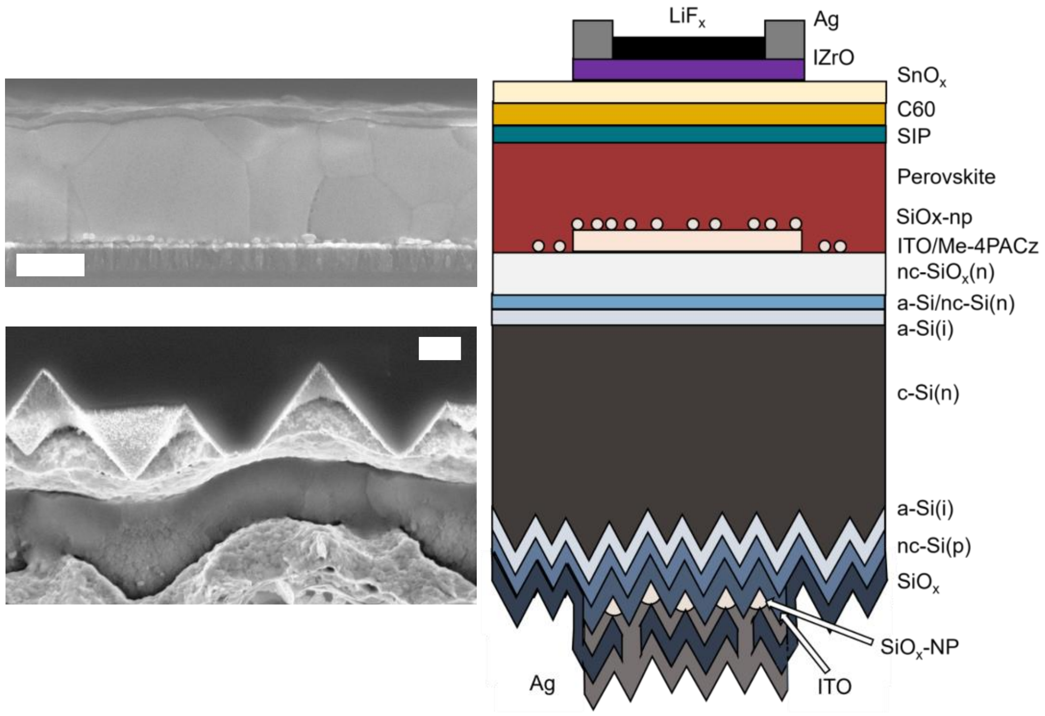

B)

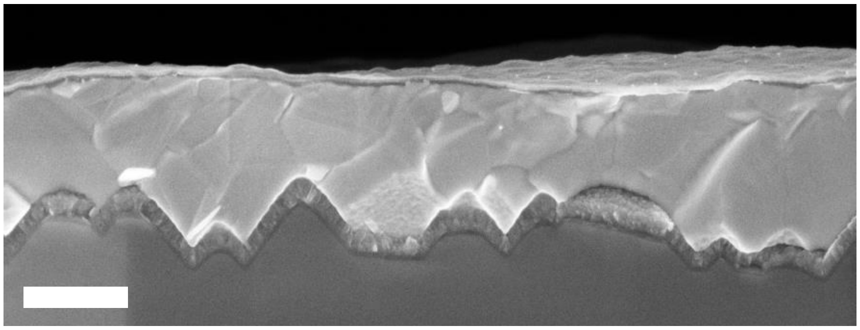

Figure S21. a) Cross-section SEM images of the top cell and the rear side of the bottom cell of the perovskite-silicon tandem device (scale bar is 300 nm for the top and 400 nm for the bottom image). b) Cross-section SEM image of the 60 cm^2 nanotextured tandems (scale bar 500 nm).

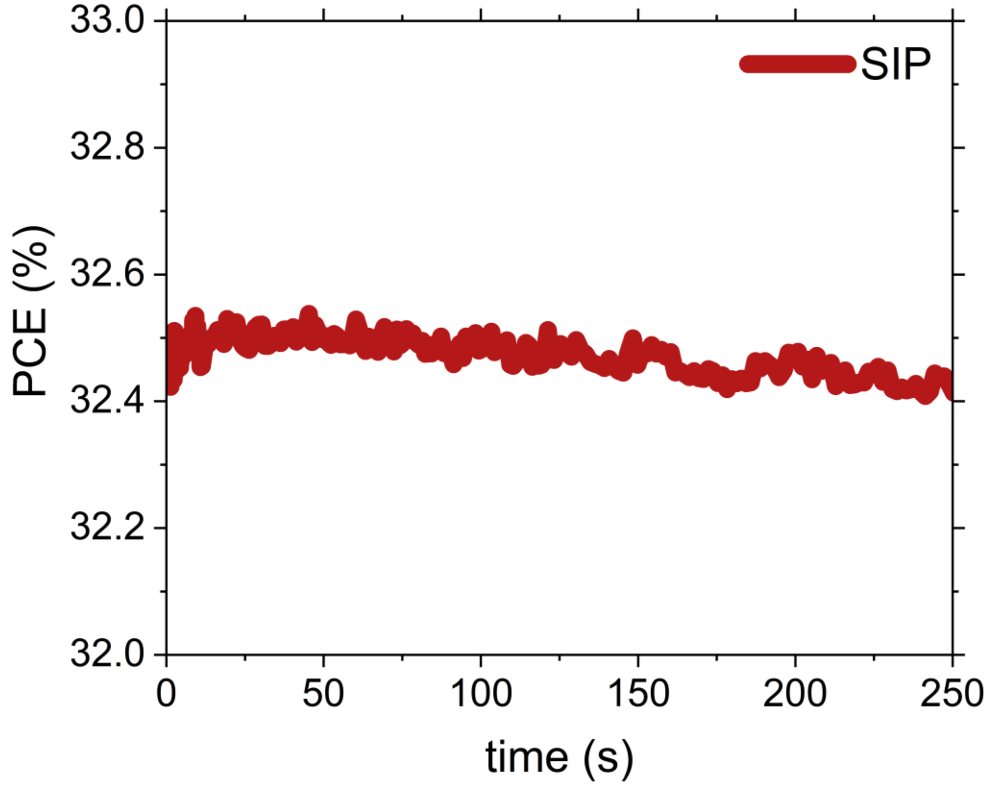

Figure S22. MPP tracking of the champion 1cm^2 devices.

Centro di Taratura
LAT N° 225
Calibration Centre
Laboratorio Accreditato di Taratura
Accredited Calibration Laboratory

ilac-MRA

ACCREDIA
L'ENTE ITALIANO DI ACCREDITAMENTO

LAT N° 225

European Commission

EUROPEAN COMMISSION
JRC - JOINT RESEARCH CENTRE
ESTI - European Solar Test Installation
Via Fermi 2749, TP450
I-21027 Ispra (Va) Italy

CERTIFICATO DI TARATURA LAT 225 DC-23-2246
Certificate of Calibration

Calibration Method: **Secondary Calibration with Reference Detector**

Procedures for temperature and irradiance corrections to measured I-V characteristics (IEC 60891, ed. 2 2009-12)
Measurement of photovoltaic current-voltage characteristics (IEC 60904-1, ed. 2 2006-09)
Measurement of current-voltage characteristics of multi-junction photovoltaic (PV) devices (IEC 60904-1-1, ed. 1 2017-05)
Requirements for photovoltaic reference devices (IEC 60904-2, ed. 3 2015-01)
Measurement principles for terrestrial photovoltaic (PV) solar devices with reference spectral irradiance data (IEC 60904-3, ed. 4.0 2019-02)
Reference solar devices – Procedures for establishing calibration traceability (IEC 60904-4, ed. 2.0 2019-11)
Computation of the spectral mismatch correction for measurements of photovoltaic devices (IEC 60904-7, ed. 4.0 2019-08)
Measurement of spectral responsivity of a photovoltaic (PV) device (IEC 60904-8, ed. 3 2014-05)
Measurement of spectral responsivity of multi-junction photovoltaic (PV) devices (IEC 60904-8-1, ed. 1 2017-05)

The measurement results reported in this certificate were obtained following procedure No. M41_j, M50_e, M51_d

Instruments or measurement standards which guarantee the traceability chain
Irradiance
Reference detector for
- IV characteristics: ASP009; Calibration laboratory: ESTI; Certificate: n. DC-23-2219
- Spectral Responsivity: ASP010; Calibration laboratory: PTB; Certificate: n. 47050-PTB-20

Temperature
Reference standard type: PRT Isotech 909 sn.1244; Calibration laboratory: UKAS 0175; Certificate n. 22-02-21
Current, Voltage and Resistance
Reference standard type: Fluke 5730/5725 sn.4811502/9085020; Calibration laboratory: DAkkS D-K-15123-01-00 Certificate No sa01075137/sa01077833

Performance Data

Standard Test Conditions
(IEC TS 61836, ed. 3.0 2016-12 par. 3.4.16.5: 1000 Wm^{-2}, 25°C, AM1.5 Global):

Short Circuit Current (I_{sc}):	**23.23 mA ± 0.39 mA**
Open Circuit Voltage (V_{oc}):	**1.9474 V ± 0.0049 V**
Maximum Power (P_{max}):	**36.33 mW ± 0.69 mW**
Efficiency at STC:	**31.6 % ± 1.0 %**

Figure S23. Certified PV performance data measured by JRC-ESTI. Measured device area is 1.15 cm^2.

Centro di Taratura
LAT N° 225
Calibration Centre
Laboratorio Accreditato di Taratura
Accredited Calibration Laboratory

ACCREDIA
L'ENTE ITALIANO DI ACCREDITAMENTO
LAT N° 225

EUROPEAN COMMISSION
JRC - JOINT RESEARCH CENTRE
ESTI - European Solar Test Installation
Via Fermi 2749, TP450
I-21027 Ispra (Va) Italy

CERTIFICATO DI TARATURA LAT 225 DC-24-2318
Certificate of Calibration

Calibration Method: **Secondary Calibration with Reference Detector**

Procedures for temperature and irradiance corrections to measured I-V characteristics (IEC 60891, ed. 3 2021-10)
Measurement of photovoltaic current-voltage characteristics (IEC 60904-1, ed. 3 2020-09)
Measurement of current-voltage characteristics of multi-junction photovoltaic (PV) devices (IEC 60904-1-1, ed. 1 2017-05)
Requirements for photovoltaic reference devices (IEC 60904-2, ed. 3 2015-01)
Measurement principles for terrestrial photovoltaic (PV) solar devices with reference spectral irradiance data (IEC 60904-3, ed. 4.0 2019-02)
Reference solar devices – Procedures for establishing calibration traceability (IEC 60904-4, ed. 2.0 2019-11)
Computation of the spectral mismatch correction for measurements of photovoltaic devices (IEC 60904-7, ed. 4.0 2019-08)
Measurement of spectral responsivity of a photovoltaic (PV) device (IEC 60904-8, ed. 3 2014-05)
Measurement of spectral responsivity of multi-junction photovoltaic (PV) devices (IEC 60904-8-1, ed. 1 2017-05)
The measurement results reported in this certificate were obtained following procedure No.: M50_e, M51_f, M66_d

Instruments or measurement standards which guarantee the traceability chain

Irradiance
Reference detector for
- IV characteristics: ASP009; Calibration laboratory: ESTI; Certificate: n. DC-23-2219
- Spectral Responsivity: ASP010; Calibration laboratory: PTB; Certificate: n. 47050-PTB-20

Temperature
Reference standard type: PRT Isotech 909 sn.1244; Calibration laboratory: UKAS 0175; Certificate n. 22-02-21

Current, Voltage and Resistance
Reference standard type: Fluke 5730/5725 sn.4811502/9085020; Calibration laboratory: DAkkS D-K-15123-01-00;
Certificate No sa01230227/sa01230215

Performance Data

Standard Test Conditions
(IEC TS 61836, ed. 3.0 2016-12 par. 3.4.16.5: 1000 Wm^{-2}, 25°C, AM1.5 Global):

Short Circuit Current (I_{sc}):	**19.96 mA ± 0.24 mA**
Open Circuit Voltage (V_{oc}):	**2.0006 V ± 0.0050 V**
Maximum Power (P_{max}):	**32.19 mW ± 0.48 mW**
Efficiency (η):	**31.11 % ± 0.92%**

JRC.C.2-ESTI E117_v e-mail: JRC-ESTI-SERVICES@ec.europa.eu

Figure S24. Second round of certification, PV performance data measured by JRC-ESTI. Measured device area is 1.035 cm^2. Certified open-circuit voltage is 2.0006V.

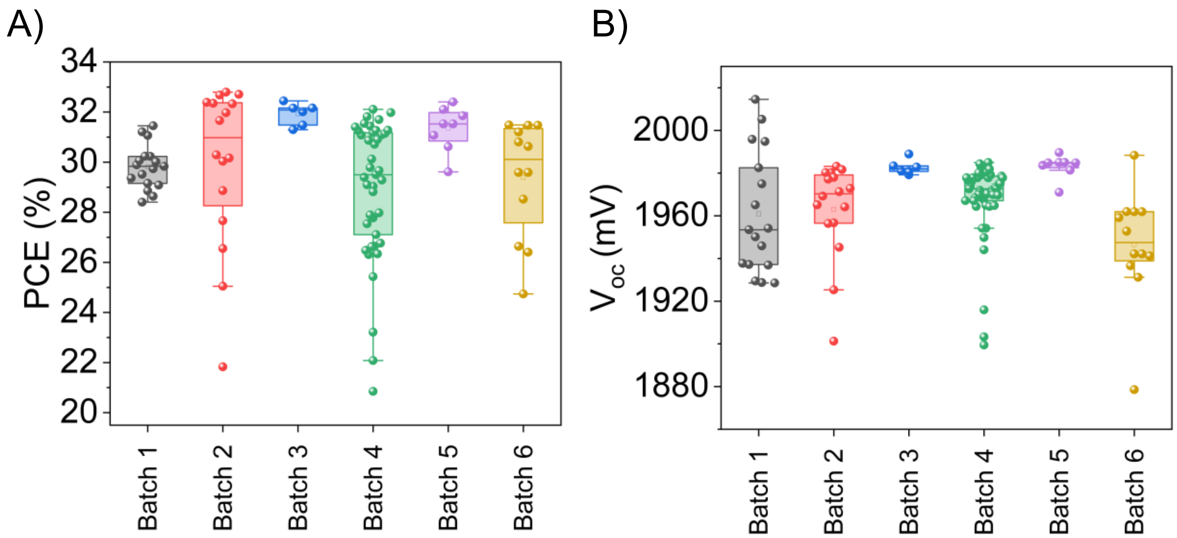

Figure S25. Device performance – a) PCE, b) V_{OC}, of perovskite-silicon tandem devices with SIP for six different batches (both reverse and forward scan included). The thickness and bandgap of the perovskite absorber differ slightly batch to batch (550-650 nm and 1.63 to 1.67 eV).

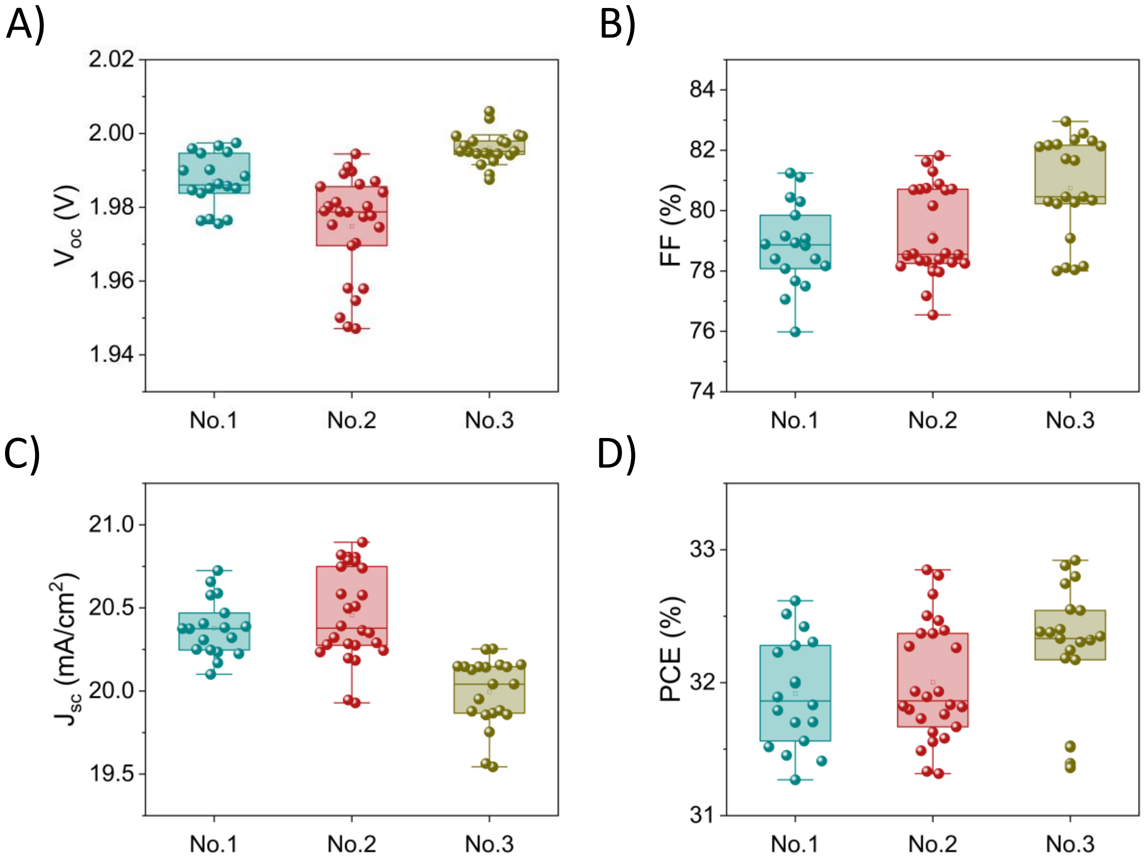

Figure S26. Device performance – a) V_{OC}, b) FF, c) J_{SC}, d) PCE of perovskite-silicon tandem devices with SIP for the best three batches (devices with low shunt resistances are not included in the analysis). High repeatability is due to the repeatable perovskite, c-Si sub-cells and the interconnection. Almost all 1cm^2 bottom cells utilized in this work have a 3.5 to 5 ms lifetime at an injection level of 5e15 cm^{-3} delivering 705 to 715 mV open-circuit voltages at half-sun illumination intensity.

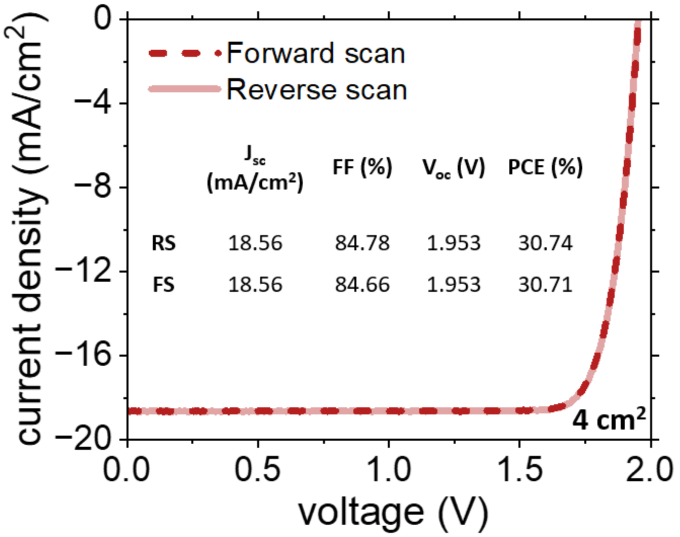

Figure S27. Reverse and forward JV curves of the champion 4 cm^2 device.

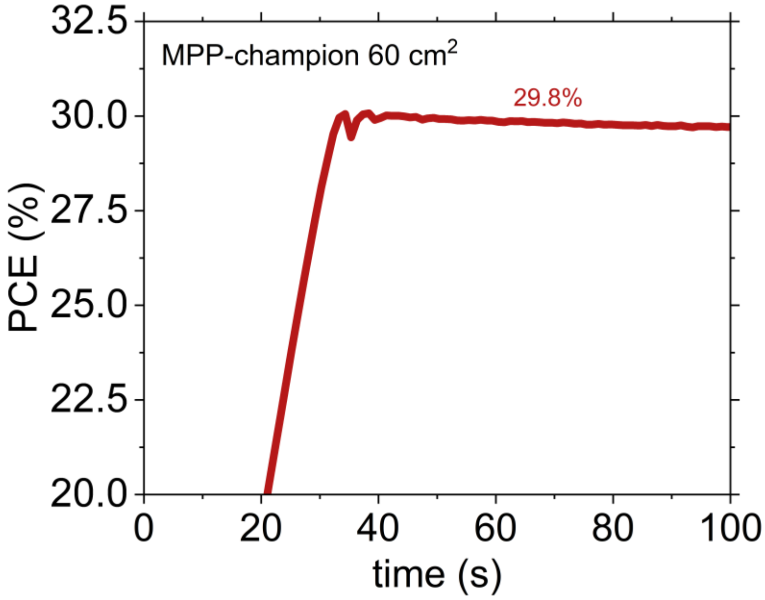

Figure S28. MPP tracking of champion 60 cm^2 device.

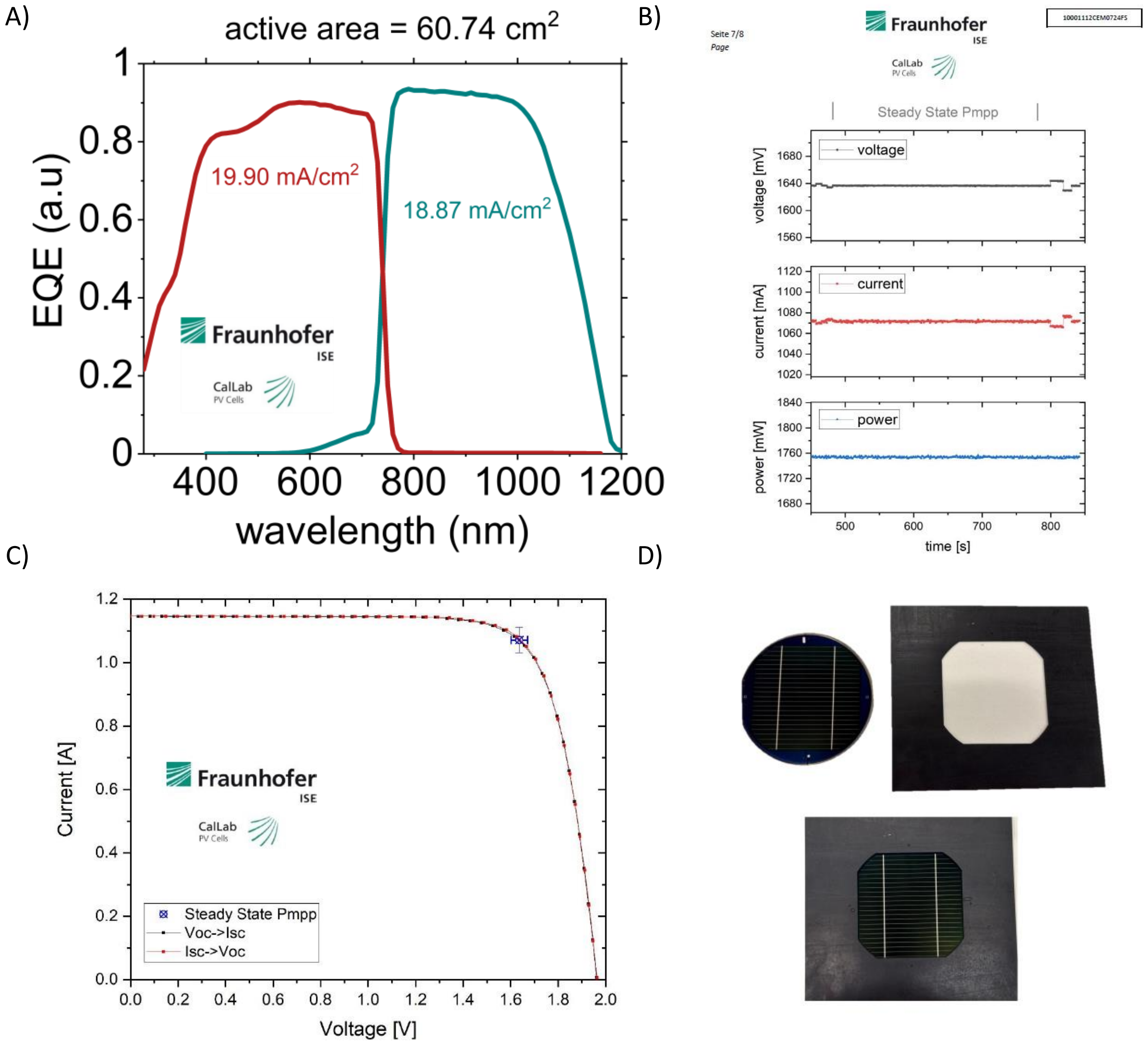

E)

		Vorwärtsrichtung / *forwards scan direction*	Rückwärtsrichtung / *reverse scan direction*	steady state MPP
V_{oc}	=	(1965 ± 20) mV	(1963 ± 20) mV	
I_{SC} (Ed.2 - 2008)	=	(1146 ± 26) mA	(1146 ± 26) mA	
I_{MPP}	=	1077 mA	1075 mA	(1072 ± 40) mA
V_{MPP}	=	1635 mV	1629 mV	(1637 ± 35) mV
P_{MPP}	=	1761 mW	1751 mW	(1754 ± 88) mW
FF	=	78.2 %	77.8 %	
η	=			(28.9 ± 1.4) %

Figure S29. Certification results from Fraunhofer ISE Callab – a) EQE, b) MPP, c) JV curve, d) masking details, e) the PV performance parameters for the 60.74 cm^2 device.

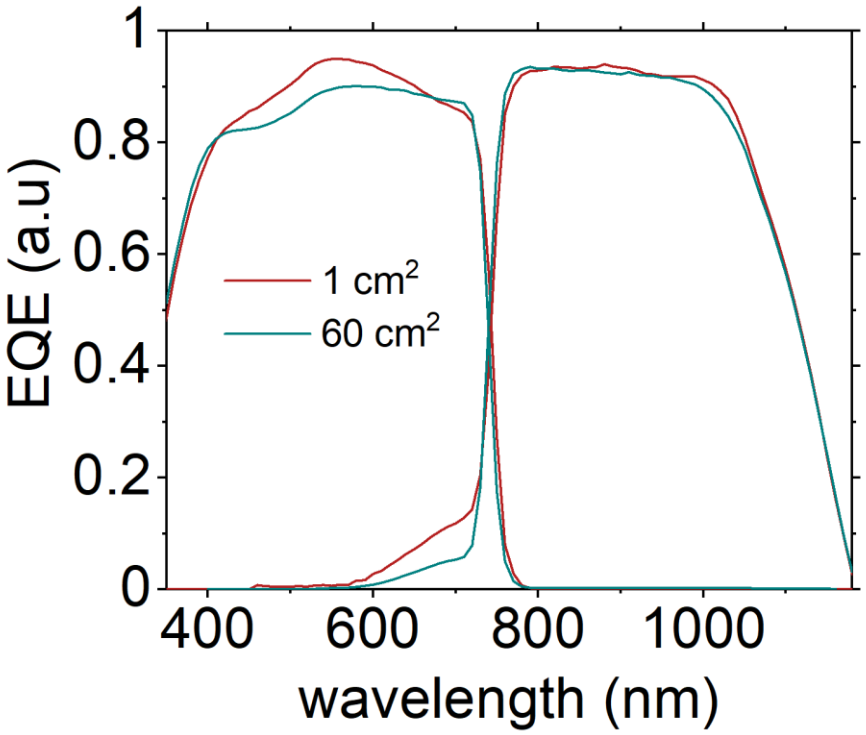

Figure S30. Comparison of the EQEs of small and large area devices. Lower response of the SHJ 60 cm^2 indicates a thicker perovskite sub-cell reducing transmitted light, but the perovskite response of the 60 cm^2 is decreased due to a thicker and less transparent top-TCO (e.g., ITO). The response from 400-550 nm is also lower in the 60 cm^2 device due to the thicker C_{60} increasing parasitic absorption. UV response from 350-400 nm is similar because 60 cm^2 devices utilize thinner ALD-SnOx from different ALD-tool (125 cycles in Oxford ALD instead of 175 cycles from PICOSUN ALD) compensating increasing parasitic absorption from C_{60}.

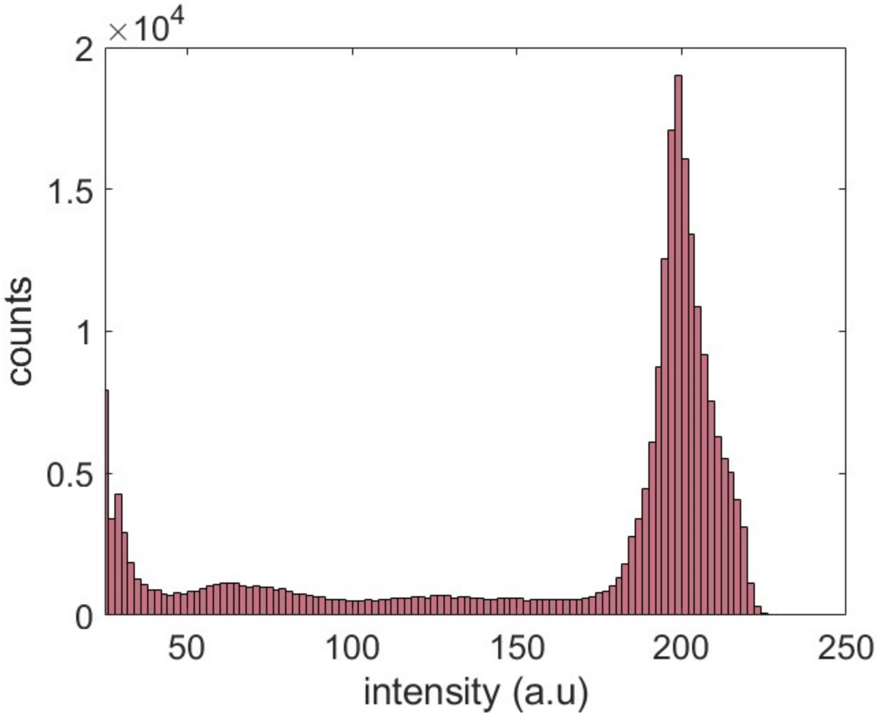

Figure S31. Histogram of the PL intensity (from the top-cell) of the PL mapping of a 60 cm^2 tandem device.

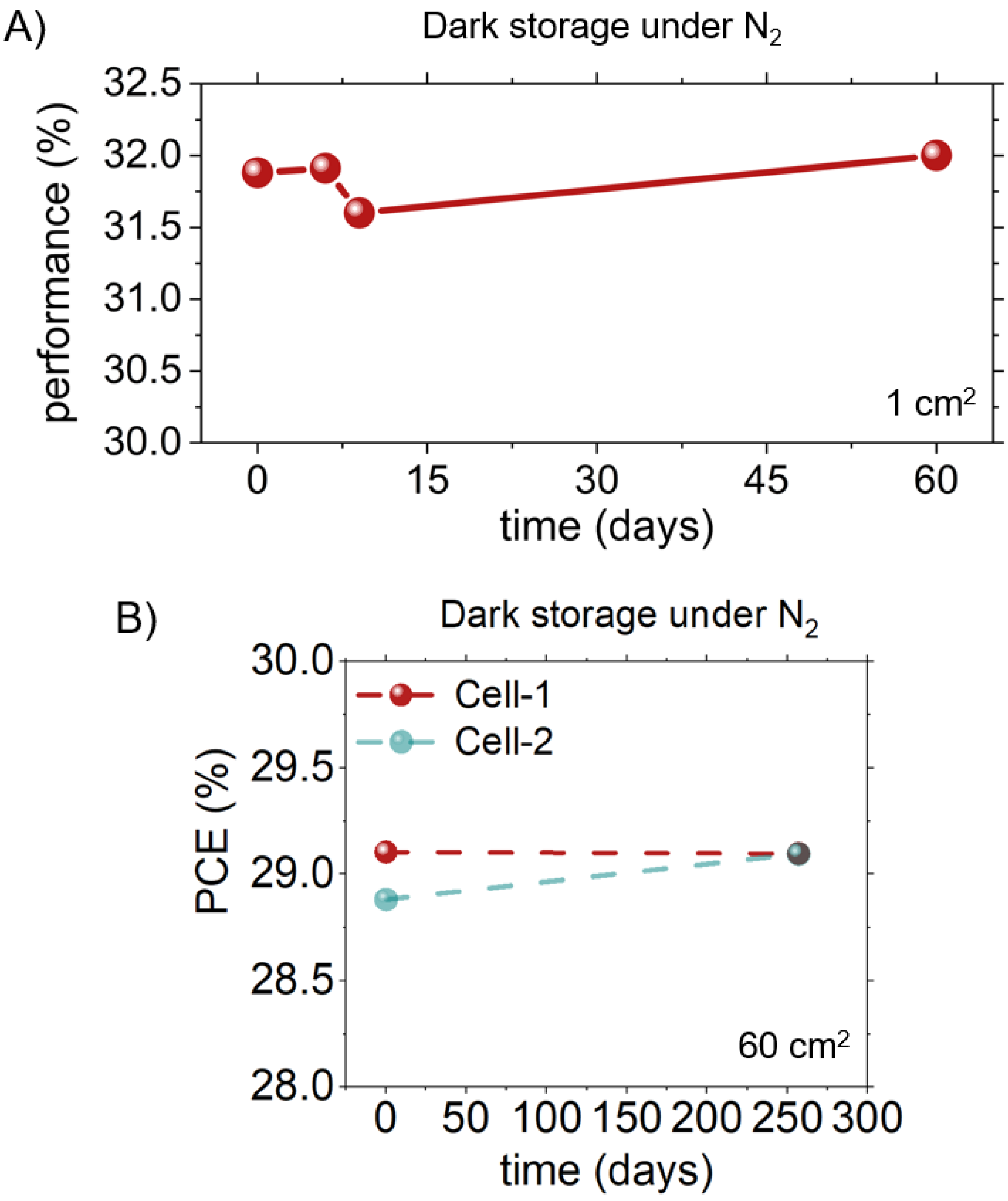

Figure S32. a) Shelf (dark and N_2) storage stability of SIP tandem device (1 device, 1 cm^2) with initial efficiency 31.88% for 60 days and b) Two 60 cm^2 devices 257 days.

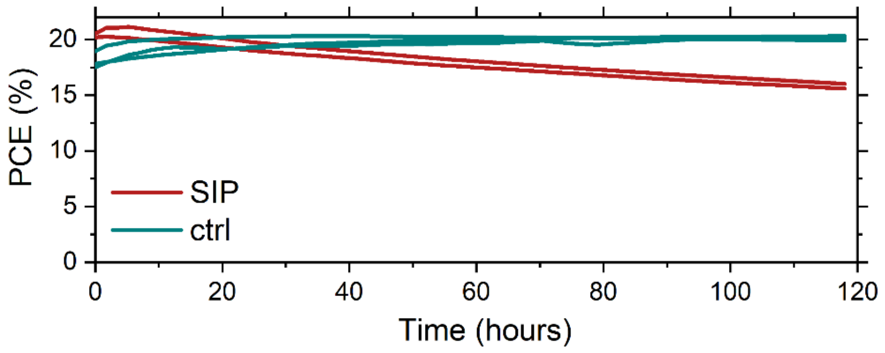

Figure S33. Maximum power point tracking of single junction devices at 35°C in N_2 without encapsulation – control (three devices) and SIP (two devices, average T_{80} lifetime of 65 hours).

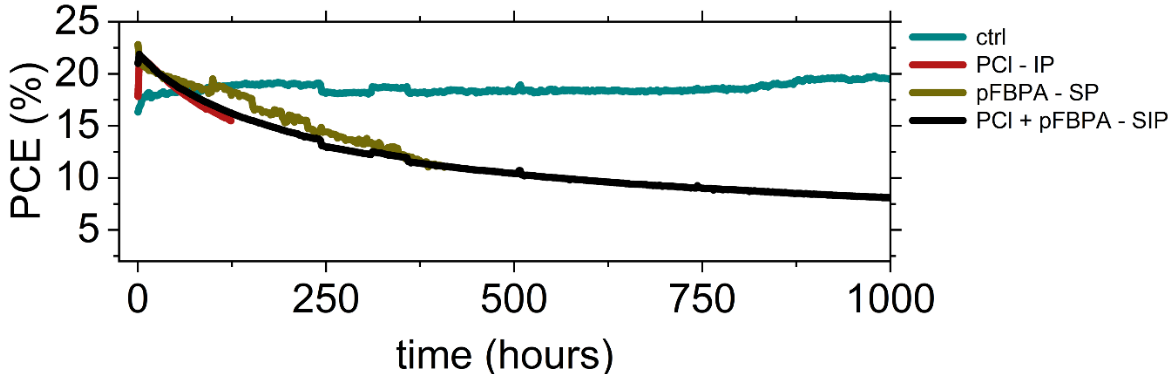

Figure S34. Maximum power point tracking of single junction devices at 35°C in N_2 without encapsulation with different surface treatments – IP, SP, SIP (one sample each condition).

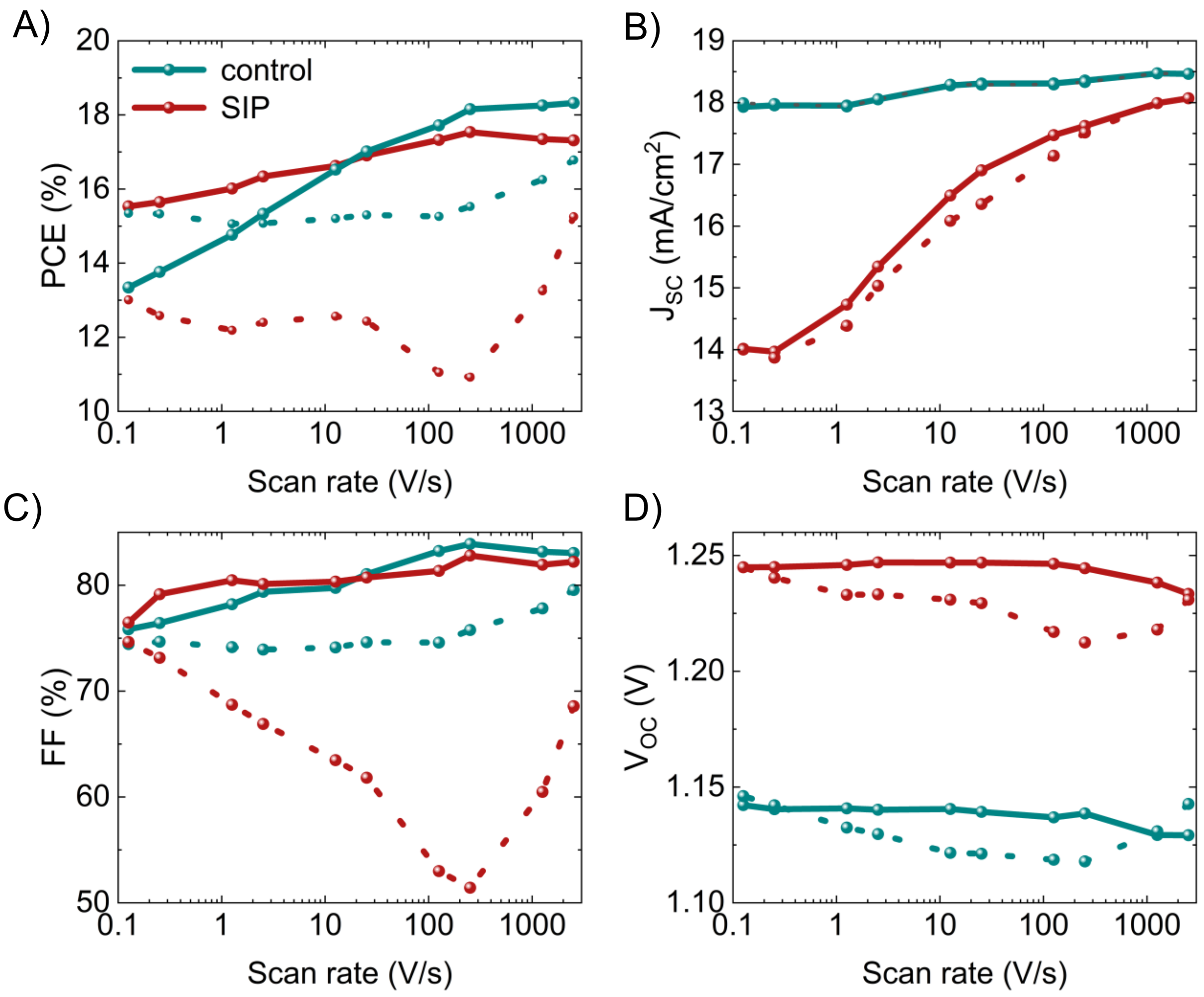

Figure S35. The a) PCE, b) Jsc, c) FF and d) Voc of control and SIP devices obtained from JV curves at different scan rates.

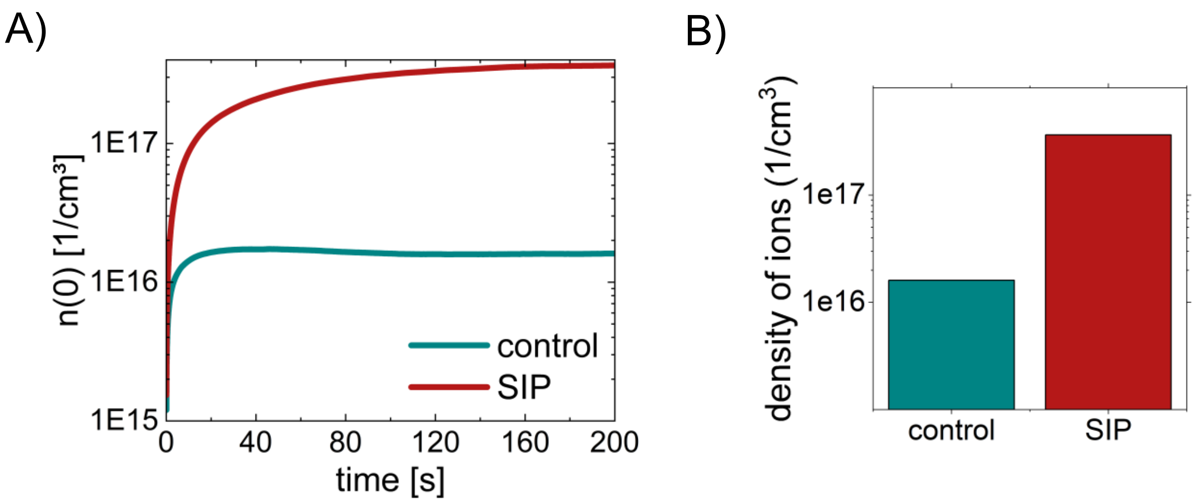

Figure S36. a) Bias-assisted charge extraction measurements to estimate the change in the density of ions, and b) estimated density of ions.

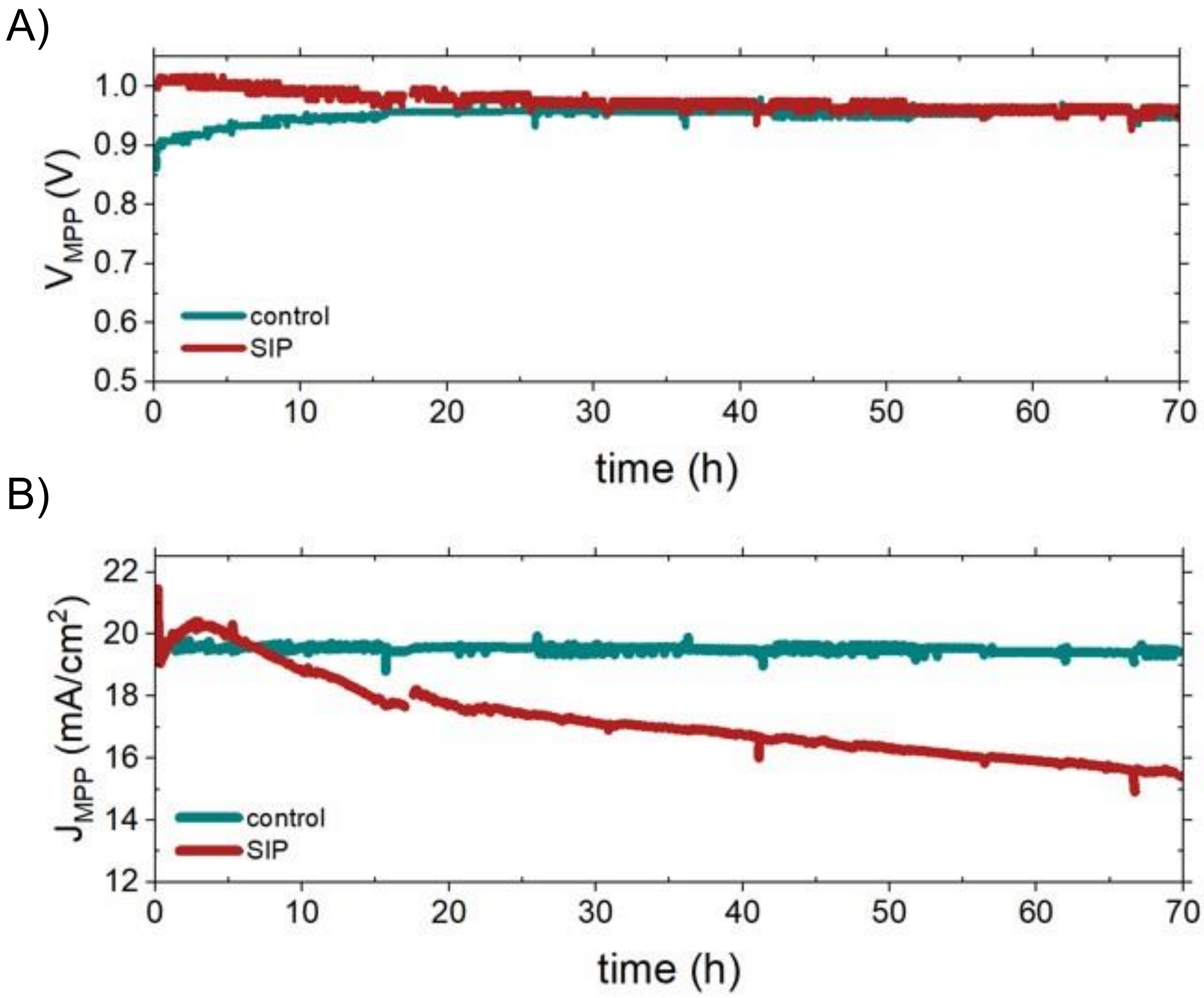

Figure S37. a) V_{MPP} and b) J_{MPP} over time at 35°C in N_2 without encapsulation – a control and a SIP device

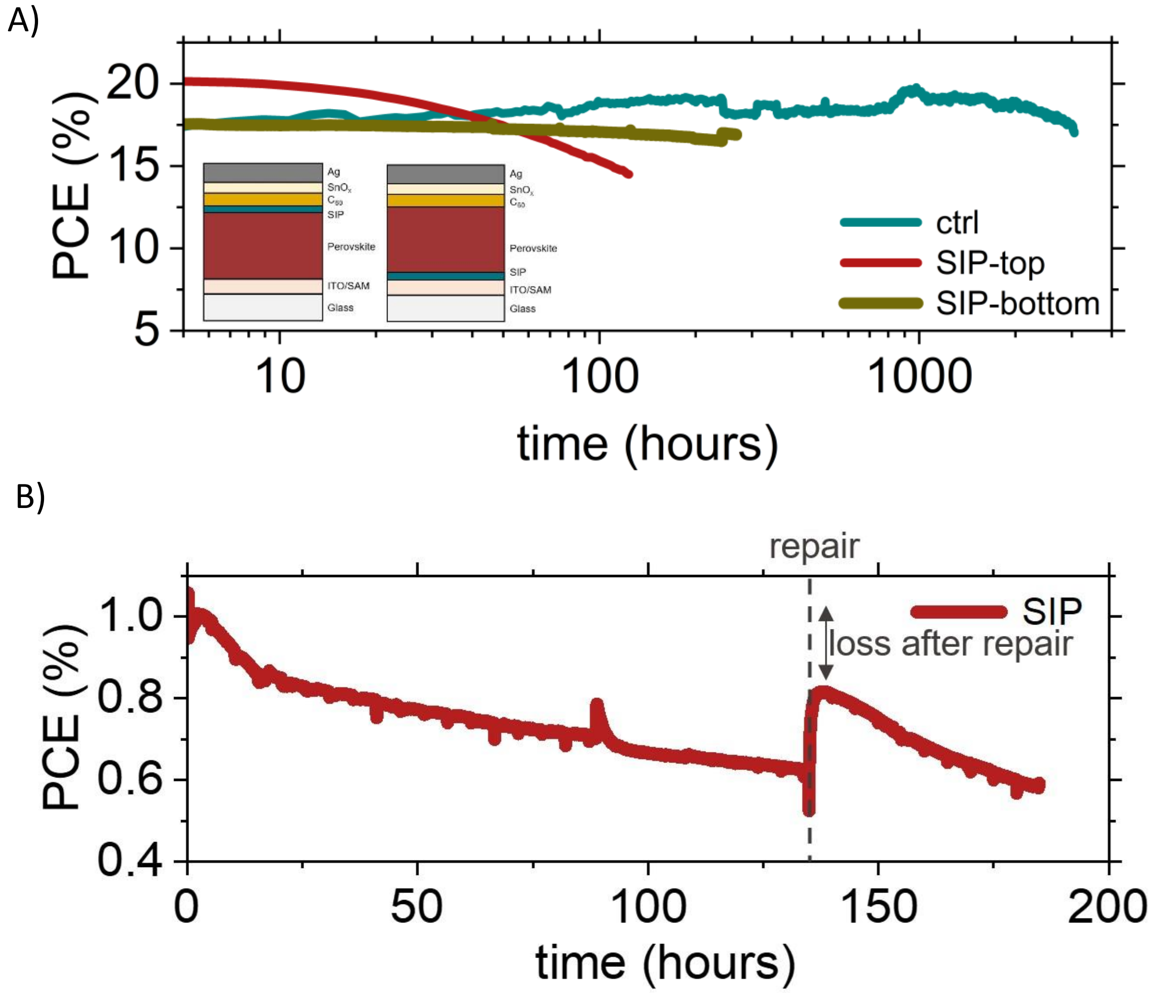

Figure S38. a) MPP tracking of the champion devices from control, SIP-top and SIP-bottom conditions under N_2 at 35 °C. b) MPP tracking of an SIP device where the top-stack is repaired after a certain time and stressed again under MPP conditions.

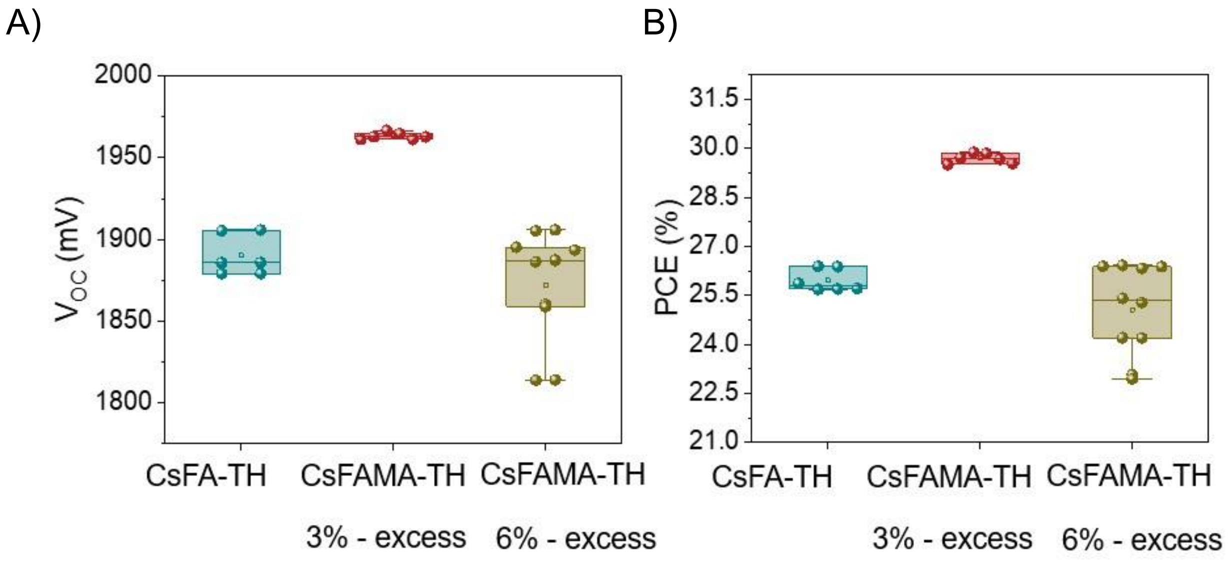

Figure S39. a) Open-circuit voltage and b) efficiency of 60 cm^2 devices with CsFA-TH (only FABr & $PbBr_2$ as Br source) and CsFAMA-TH (FABr, MABr and $PbBr_2$ as Br source) absorbers. Due to the curing (130°C, 10 min, in ambient) after screen-printing, CsFA-TH absorbers degraded delivering lower performance (<27%).
