# Supplementary material for: 60 cm2 perovskite-silicon tandem solar cells with an efficiency of 28.9% by homogeneous passivation
Source: Nat Commun. 2025 Sep 30;16:8672. doi: 10.1038/s41467-025-63673-y (PMC12485175; doi:10.1038/s41467-025-63673-y)
Supplement: Supplementary file 2 — Reporting Summary [file 41467_2025_63673_MOESM2_ESM.pdf]

nature portfolio

Corresponding author(s): Kerem Artuk and Dr. Christian Wolff

# Solar Cells Reporting Summary

Nature Portfolio wishes to improve the reproducibility of the work that we publish. This form is intended for publication with all accepted papers reporting the characterization of photovoltaic devices and provides structure for consistency and transparency in reporting. Some list items might not apply to an individual manuscript, but all fields must be completed for clarity.

For further information on Nature Research policies, including our data availability policy, see Authors & Referees.

## Experimental design

**Please check the following details are reported in the manuscript, and provide a brief description or explanation where applicable.**

1. Dimensions

| Item | Yes/No | Description |
|---|---|---|
| Area of the tested solar cells | ☒ Yes ☐ No | 1 cm2, 4cm2 and 60.74cm2<br>*Explain why this information is not reported/not relevant.* |
| Method used to determine the device area | ☒ Yes ☐ No | Laser-cut metal (for single junction perovskite) and rubber masks (for perovskite-Si tandems) (with a specified accuracy of around 0.01 mm are used). The dimensions are verified with an optical microscope.<br>*Explain why this information is not reported/not relevant.* |

2. Current-voltage characterization

| Item | Yes/No | Description |
|---|---|---|
| Current density-voltage (J-V) plots in both forward and backward direction | ☒ Yes ☐ No | Details in Methods (JV) section. Example figures, Fig.3(a) and Fig.3(f). |
| Voltage scan conditions | ☒ Yes ☐ No | Details in Methods (JV) section. Example figures, Fig.3(a) and Fig.3(f).<br>*Explain why this information is not reported/not relevant.* |
| Test environment | ☒ Yes ☐ No | For perovskite-Si tandem solar cells - temperature controlled (25 ºC) chuch, in ambient. For single-junction perovskite solar cells, no temperature control, in ambient.<br>*Explain why this information is not reported/not relevant.* |
| Protocol for preconditioning of the device before its characterization | ☐ Yes ☒ No | *Provide a description of the protocol.*<br>No preconditioning utilized. |
| Stability of the J-V characteristic | ☒ Yes ☐ No | MPP tracking for the champion devices are provided, Fig.S20-26. In certifications, also maximum power point tracking is provided, we can include in SI if needed.<br>*Explain why this information is not reported/not relevant.* |

3. Hysteresis or any other unusual behaviour

| Item | Yes/No | Description |
|---|---|---|
| Description of the unusual behaviour observed during the characterization | ☐ Yes ☒ No | *Provide a description of hysteresis or any other unusual behaviour observed during the characterization.*<br>Not observed, with the scan rates reported for tandems (0.1V/s), hysteresis is negligible - Fig.3(a) and Fig.3(f). |
| Related experimental data | ☒ Yes ☐ No | Not observed, with the scan rates reported for tandems (0.1V/s), hysteresis is negligible - Fig.3(a) and Fig.3(f).<br>*Explain why this information is not reported/not relevant.* |

4. Efficiency

| Item | Yes/No | Description |
|---|---|---|
| External quantum efficiency (EQE) or incident photons to current efficiency (IPCE) | ☒ Yes ☐ No | Absolute EQE measurements have been utilized using calibrated Gold Si cell from ISFH.<br>*Explain why this information is not reported/not relevant.* |

A comparison between the integrated response under the standard reference spectrum and the response measure under the simulator

☒ Yes ☐ No

Fig.3(a) and Fig.3(d) gives a comparison between in-house EQE and JV. For each certification report, there are also EQE and IV (e.g., Fig.S(29))

*Explain why this information is not reported/not relevant.*

For tandem solar cells, the bias illumination and bias voltage used for each subcell

☒ Yes ☐ No

For the top-cell, 0.7V and IR light bias. For the bottom-cell, 1.2V and blue light bias.

*Explain why this information is not reported/not relevant.*

## 5. Calibration

Light source and reference cell or sensor used for the characterization

☒ Yes ☐ No

Calibrated Si cells from Fraunhofer ISE (red, blue and black) in JV and Calibrated Gold cell from ISFH in EQE.

*Explain why this information is not reported/not relevant.*

Confirmation that the reference cell was calibrated and certified

☒ Yes ☐ No

Fraunhofer ISE and ISFH

*Explain why this information is not reported/not relevant.*

Calculation of spectral mismatch between the reference cell and the devices under test

☒ Yes ☐ No

1.009 for the top cell, and 1.000 for the bottom

*Explain why this information is not reported/not relevant.*

## 6. Mask/aperture

Size of the mask/aperture used during testing

☒ Yes ☐ No

1 cm2, 4cm2 and 60.74cm2.

*Explain why this information is not reported/not relevant.*

Variation of the measured short-circuit current density with the mask/aperture area

☐ Yes ☒ No

*Report the difference in the short-circuit current density values measured with the mask and aperture area.*

*Explain why this information is not reported/not relevant.*

## 7. Performance certification

Identity of the independent certification laboratory that confirmed the photovoltaic performance

☒ Yes ☐ No

Fraunhofer ISE Cal Lab for 60cm2 cell and JRC-ESTI for 1cm2 cells

*Explain why this information is not reported/not relevant.*

A copy of any certificate(s)

☒ Yes ☐ No

Fig.S(23,24,29) - three certificates.

*Explain why this information is not reported/not relevant.*

## 8. Statistics

Number of solar cells tested

☒ Yes ☐ No

Fig.3(c,e) and Fig.S(23,24)

*Explain why this information is not reported/not relevant.*

Statistical analysis of the device performance

☒ Yes ☐ No

Fig.3(c,e) and Fig.S(25,26)

*Explain why this information is not reported/not relevant.*

## 9. Long-term stability analysis

Type of analysis, bias conditions and environmental conditions

☒ Yes ☐ No

Fig.S(33,34) - MPP tracking in N2 at 35ºC - unencapsulated devices with 0.2 cm2 active area with 0.1 cm2 shadow mask - we can provide schematics if needed.

*Explain why this information is not reported/not relevant.*
